# Characterizing the Conformational Landscape of Flavivirus Fusion Peptides via Simulation and Experiment

**DOI:** 10.1038/srep19160

**Published:** 2016-01-20

**Authors:** Jan K. Marzinek, Rajamani Lakshminarayanan, Eunice Goh, Roland G. Huber, Sadhana Panzade, Chandra Verma, Peter J. Bond

**Affiliations:** 1 National University of Singapore, Department of Biological Sciences, 14 Science Drive 4, Singapore 117543; 2 Bioinformatics Institute (A*STAR), 30 Biopolis Str., #07-01 Matrix, Singapore 138671; 3 Singapore Eye Research Institute, The Academia, 20 College Road, Discovery Tower Level 12, Singapore 169856; 4 School of Biological Sciences, Nanyang Technological University, 60 Nanyang Drive, Singapore 637551.

## Abstract

Conformational changes in the envelope proteins of flaviviruses help to expose the highly conserved fusion peptide (FP), a region which is critical to membrane fusion and host cell infection, and which represents a significant target for antiviral drugs and antibodies. In principle, extended timescale atomic-resolution simulations may be used to characterize the dynamics of such peptides. However, the resultant accuracy is critically dependent upon both the underlying force field and sufficient conformational sampling. In the present study, we report a comprehensive comparison of three simulation methods and four force fields comprising a total of more than 40 μs of sampling. Additionally, we describe the conformational landscape of the FP fold across all flavivirus family members. All investigated methods sampled conformations close to available X-ray structures, but exhibited differently populated ensembles. The best force field / sampling combination was sufficiently accurate to predict that the solvated peptide fold is less ordered than in the crystallographic state, which was subsequently confirmed via circular dichroism and spectrofluorometric measurements. Finally, the conformational landscape of a mutant incapable of membrane fusion was significantly shallower than wild-type variants, suggesting that dynamics should be considered when therapeutically targeting FP epitopes.

Flaviviruses are one of the main causative agents of infectious disease and deaths in the world^1^. Flavivirus members form a genus within the *Flaviviridae* family, and include Dengue (DENV), tick-borne encephalitis (TBE), west nile virus (WNV), Japanese encephalitis (JEV), yellow fever (YFV) and Saint Louis encephalitis (SLEV)^2^,^3^,^4^. Currently there are no approved drugs or vaccines that are effective against WNV, SLEV, or against all DENV serotypes^5^,^6^ though several vaccine candidates have been tested^7^,^8^,^9^,^10^. The icosahedral flavivirus particle contains a positive, single-stranded RNA genome surrounded by the capsid protein, along with the membrane and envelope (E) proteins. The initial infection process^11^,^12^ involves receptor-mediated endocytosis of the virus and exposure to lowered pH. This results in a marked structural rearrangement in the E proteins^13^, leading to exposure of the hydrophobic tips of the fusion peptide (FP) stretching from residue numbers 98 to 112 in domain II of the E protein, which subsequently interacts with the host membrane initiating fusion.

The FP adopts a ~15 amino acid long hairpin-like conformation, which contains a central turn around residue 104, and its sequence is highly conserved, varying by at most two amino acid substitutions among all known members of the flavivirus family. A wide body of medium to high resolution structural data is available for wild-type FP variants. The FP of Dengue serotypes 2 and 4 (DENV-2, DENV-4) has the sequence: D^98^RGWGNGCGLFGKGG^112^ (referred to as FP-1 throughout this text)^14^. The envelope protein FP of the two remaining Dengue serotypes (DENV-1, DENV-3) along with WNV, JEV, YFV, and SLEV has a single amino acid substitution (G112S) compared to FP-1, with sequence: D^98^RGWGNGCGLFGKGS^112^ (referred to here as FP-2)^14^. The FP from TBE has one further amino acid substitution relative to FP-2 (G104H)^14^, with sequence: D^98^RGWGNHCGLFGKGS^112^ (referred to here as FP-3). Structural alignment of available FP X-ray crystal structures from all flaviviruses (Fig. 1) reveals that despite several amino acid substitutions and alternative states, almost identical folds are adopted, with a maximum pair-wise Cα root-mean square deviation (RMSD) of <0.08 nm. The X-ray structures of the FP in both pre-fusion and post-fusion conformations reveal two exposed aromatic side chains (W101, F108) arranged in close proximity (Fig. 1) forming a hydrophobic patch at the tip of the loop, that has been hypothesised to be responsible for penetration of the host cell membrane^15^.

The two aromatic residues have been shown to play a critical role in viral infectivity^12^,^16^,^17^. A solution nuclear magnetic resonance (NMR) structure of FP-1 bound to a dodecylphosphocholine (DPC) micelle revealed a similar hydrophobic patch, in this case formed by a hydrophobic triad that included, in addition to the aromatic residues, reoriented L107^12^. Fluorescence spectroscopy also revealed that the W101A mutation relative to FP-1, with sequence: D^98^RGAGNGCGLFGKGG^112^ (referred to as FP-4 throughout this text), completely abolishes the binding of FP to lipid bilayers^12^. Interestingly, crystal structures of the whole E protein in either mature, immature or post-fusion conformation reveal a short 3_10_-helix motif for the three FP residues W101-N103, immediately preceding the central turn. On the other hand, circular dichroism (CD) measurements previously suggested that a shortened 13-residue peptide with the sequence D^98^RGWGNGCGLFGK^110^ (corresponding to residues 98-110 from all flavivirus FPs except TBE) may adopt a less ordered structure lacking the helical motif^18^, which is consistent with NMR data^12^.

The structure of FP may vary depending upon its local environment and/or mechanistic states. Moreover, different flavivirus sequences and mutants are crucial in determining membrane fusion ability and hence propensity for viral infection. The high degree of conservation of FP across the flavivirus family means that it constitutes an attractive target for antiviral drugs^12^,^13^,^19^. Several antibodies^20^,^21^ and small molecule inhibitors^2^ are known to bind FP. It is thus important to understand the conformational landscape associated with the FP, in order to rationalize therapeutic targeting of this region.

Molecular dynamics (MD) simulations represent a powerful tool for studying the preferred structure/fold and associated conformational dynamics of such peptides at atomic-scale resolution, providing data which can support and extend those from experimental methods^22^,^23^,^24^,^25^,^26^. However, the accuracy of MD is critically dependent both upon the force field (FF), which describes the underlying forces and energies of interaction within the system of interest, as well as the simulation sampling, which determines what biologically relevant timescales are accessible. A range of highly optimized FFs are available for peptides, proteins, and other biomolecules. Recently, the accuracy of different FFs has been assessed for several peptides and proteins using conventional MD simulation sampling approaches^27^,^28^,^29^,^30^,^31^, and in some cases focused on improving FFs and/or comparing with experimental data^32^,^33^,^34^,^35^,^36^. Likewise, alternative MD simulation approaches have been developed which can in principle overcome limitations associated with sampling the conformational landscape of a given molecular system^38^. For example, replica exchange MD (REMD) is an enhanced sampling method in which a number of system replicas evolve independently at a range of temperatures, whilst exchanging coordinates at specified time intervals according to the Metropolis criterion^39^. Simulated annealing (SA) represents another approach to improve conformational sampling, in which the simulation system is alternatively heated and cooled within specific temperature ranges for a given number of cycles^40^.

A present day challenge in peptide simulations thus lies in making a suitable choice of sampling methodology as well as FF. However, the efficiency and/or accuracy of different sampling methods in the context of multiple FFs is yet to be fully assessed. This is further complicated in the case of short peptides with limited/variable well-defined secondary structural regions such as FP, for which FFs are still being evaluated and refined^25^. In this work, we have employed combinations of various simulation methods and FFs (Table 1), comprising a total of over 40 μs of sampling in explicit solvent, in order to explore the conformational space accessible to all three FP variants from the flavivirus family, along with the disruptive W101A mutant. Given the differences in FP secondary structural content reported based on various experimental approaches and constructs, we first performed CD and spectrofluorometry experiments for FP-1. The results suggested that the dominant FP conformation in solution does not contain ordered secondary structure; a more ordered conformation containing a short central 3_10_-helix may exist only in the crystallographic state. We subsequently explored the conformational dynamics of FP-1 and characterized its complete free-energy landscapes using conventional MD, REMD, and SA, with four widely used force fields, Amber99SB*-ILDN-Q^41^, CHARMM36^42^, Gromos54A7^43^, OPLS-AA^44^. For comparison, we also compared the older CHARMM22/CMAP^45^,^46^ forcefield with its improved CHARMM36 counterpart. After confirming that structural and dynamic convergence had been achieved, we assessed the “structural accuracy” of these FF and method combinations by comparing dominant sampled backbone conformations to available X-ray structures. As expected, consistent with our spectroscopic analysis, the central 3_10_-helix observed across crystal structures was either transient or not observed in the solvated state. Nevertheless, W101 and F108 tended to form a “hydrophobic core” that biases the peptide towards the experimentally observed hairpin-like fold. All methods employed were shown to sample backbone conformations close to the experimental structures although with significantly different relative populations. However, large differences in performance were observed across FFs. The CHARMM36 and in particular Amber FF in combination with REMD best generated a well-populated hairpin-like fold which closely resembled the crystallographic state, and comparable accuracy was subsequently achieved using Amber for the FP-2, FP-3, and W101A FP-4 variants. Finally, whilst FP-4 adopts an equilibrium backbone conformation comparable to the wild-type flavivirus family variants, its free-energy landscape is significantly shallower than that of the other FP constructs, and explores more open or unfolded conformations. Thus, the loss of infective activity in the W101A mutant may be due to the disruption of the conformational landscape in the highly conserved FP hairpin-like structure. This also suggests that the dynamics of wild-type FP variants, and by analogy unstructured viral peptides in general, should be considered when developing drugs or antibodies targeted at inhibiting the fusion mechanism.

## Results

### Spectroscopic Assessment of the FP-1 Fold

Crystal structures of flavivirus FP reveal a short 3_10_–helix (W101-N103) within the loop, immediately preceding the central turn, whereas CD measurements indicate a completely unstructured coil for shorter FP fragments^18^. To examine this further, we conducted CD experiments on the full-length FP-1 sequence. The corresponding spectrum is presented in Fig. 2B, and indicates that FP-1 adopts a random coil conformation (wavelength 190–200 nm), consistent with earlier findings, suggesting that the 3_10_–helix element may occur only under crystallographic conditions. To further confirm our findings, we used spectrofluorometry on full length FP-1. Tryptophan serves as an excellent intrinsic protein probe due to its sensitivity to the surrounding environment, thus providing information reflecting local protein structure and associated conformational changes^47^. Due to the presence of another aromatic residue in FP-1 (F108, which is fully conserved among the flaviviruses), the spectrofluorometry analysis was conducted at two excitation wavelengths, 280 and 290 nm^48^. In both cases, the maxima of emission were observed at approximately 355 nm. At this pH, a fully exposed tryptophan residue in proteins correspond to an emission of 353 nm. In addition, hydrophobic residues are known to be more exposed in structurally disordered proteins^47^. Hence, this further lends support to the 15 residue FP-1 construct existing in solution as a random coil^49^,^50^.

### Characterization of FP-1 via Simulation

#### Assessment of Sampling Convergence and Efficiency

We first assessed the convergence of the trajectories arising from different combinations of each force field and simulation method. Block analysis was used to calculate the percentage of total sampled conformations that were required to achieve convergence^51^. This revealed that FP-1 peptide structural properties including radius of gyration, end-to-end distance, and solvent accessible surface area (SASA) had converged within 20% of the simulation sampling across all methods and FFs, though slightly greater fluctuations were observed for conventional MD consistent with the reduced rate of sampling, whilst mean values did not differ significantly from experiment (Supplementary Figs 1–4). Slightly larger values for the SASA and radius of gyration were observed for CHARMM36^42^ in comparison with other forcefields, which may be explained by its refinement against solution NMR data for weakly structured peptides. Likewise, block analysis of the backbone root mean square fluctuations (RMSFs) revealed excellent convergence, within 20–40% of the total sampling time (Supplementary Fig. 5), confirming peptide dynamics were fully sampled in each system.

Having confirmed that each method does a reasonable job of achieving structural and dynamic convergence within the total sampling times generated here, we next sought to identify the associated sampling efficiency in exploring different conformations. Thus, clustering analysis with a constant cut off of 0.45 nm was first conducted for the FP-1 construct on a pseudo trajectory comprising concatenated frames from all methods and FFs (Table 1). The sampling efficiency was assessed in terms of the fraction of the total simulation time required for each method/FF to explore a certain percentage of the total explored conformations (Fig. 3). We first assessed the sampling efficiency of REMD, SA, and MD, irrespective of FF, before subsequently assessing it for different FFs irrespective of method.

In total, the combined trajectory of all methods and FFs explored a total of 24 clusters. Interestingly, when comparing the methodologies employed, it may be noted that SA and REMD explored 23 out of 24 clusters in comparison to MD with one cluster less (22 out of 24). As shown in Fig. 3, each method required almost the total simulation time to converge to 100% of its own explored conformations (~90% for REMD, ~85% for SA and 95% for MD). However, that corresponded to ~13,000 ns for REMD and ~4,000 ns for SA and MD. Whilst 50% of the conformations were explored rapidly within <10% of the computational time for each method, REMD required approximately ten times longer total simulation time (~1,000 ns for REMD versus 100 ns for MD and SA). In order to explore 80% of conformations, MD converged the fastest (11% of the total simulation time) in comparison to REMD and SA (24 and 21% of the total simulation time respectively). The fastest convergence of 30% of all conformations was achieved by SA resulting in 0.3% of the total simulation time, which may be explained by the major shifts in phase space that are sampled by this method by fast heating/cooling cycles. Convergence in exploration of clusters confirms that the results from each method/FF were unbiased by the starting structure. In particular, conventional MD is typically most likely to become trapped in local minima, but explored a comparable number of total clusters as REMD and SA within a fraction of the total sampling (Fig. 3).

When comparing FFs, it may be observed that Amber and Gromos explored the highest number of all conformations (22 and 23 clusters out of 24), which is slightly higher than in OPLS-AA (20 clusters) or CHARMM36 (21 clusters). 30% of all conformations were explored within 1% of total calculation time by Gromos, CHARMM36 and OPLS-AA. In contrast, Amber required 3% of the same time. Similarly that was the case for 50% of conformations where Amber required ~10% whereas remaining FFs needed ~3% of the total sampling. In contrast, complete exploration of all conformations was achieved within approximately 50% and 63% of the total calculation time for Amber and CHARMM36 respectively, whilst OPLS-AA and Gromos required nearly 80–90% of the total sampling. Likewise, Amber was initially slower to sample conformations (with 30% of clusters reached within ~170 ns, compared to ~60–80 ns for the other FFs). Hence, Amber seems to be less efficient at sampling “rare conformations” of the phase space. For all FFs, 80% of conformations were explored within <30% of the total calculation simulation time. The application of the same clustering protocol on single trajectories corresponding to a given method/FF provided 7 to 21 clusters depending upon the combination of FF and sampling methodology employed (Table 2). REMD clusters were short-lived and not continuously present over the whole simulation time, as shown in Supplementary Figs 6–10 for all the FFs. On the other hand, in the case of conventional MD, the first few most populated conformations were independently and less frequently sampled than for REMD, where they were well represented throughout the simulation. For example, with Amber (Supplementary Fig. 6) the first and second clusters oscillate with one another over periods of approximately hundreds of nanoseconds throughout the simulation. Similar observations may be noted for the remaining FFs (Supplementary Figs 7–10), and in general support the notion that even relatively dynamic, unstructured peptides may become trapped within different local minima on the energy landscape, necessitating microsecond timescales to properly sample such systems when utilizing conventional MD. In contrast, several of the most populated configurations remained persistent when using either of the enhanced sampling methods.

Figure 4 shows network analysis of the generated clusters, illustrating how different methods and force fields sample conformational space. The colour of nodes corresponds to the respective node population. Hence, it is apparent how the individual approaches differ in sampling conformational space for the fusion peptide. Simulated annealing covers the conformational space more equitably than either REMD or MD as indicated by the generally more uniform colour distribution across the nodes. Conformational sampling in REMD simulations is generally comparable to distributions observed by conventional MD. The Amber and Gromos force fields are largely similar in their conformational diversity whereas CHARMM36^42^ exhibits increased sampling in the rarer states. The OPLS-AA force field shows higher occupancy of the first cluster compared to the other investigated force fields. Equivalent time progression series of the conformational networks at 20%, 40%, 60%, 80% and 100% of full simulation time for each method and force field are provided in Supplementary Figs 11–17.

#### Structural Analysis

**Figure 1:**
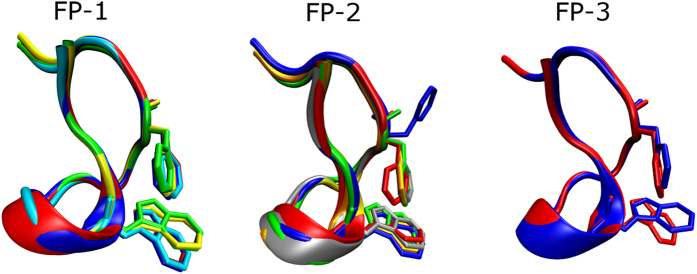
Structural alignment of all known crystal structures for the three peptides considered in this study. These consist of: FP-1 – 1OAN (blue), 1OK8 (yellow), 1TG8 (red), 3C5X (green), 3UAJ (cyan); FP-2 – 3G7T (blue), 1UZG (red), 2HG0 (silver), 2I69 (orange), 3I50 (yellow), 4FG0 (green); FP-3 – 1SVB (blue), 1URZ (red). The protein fold is represented as cartoons, with the two key aromatic side chains of W101 and F108 shown in wireframe format. Details of the alignment protocol can be found in the Methods section.

**Figure 2:**
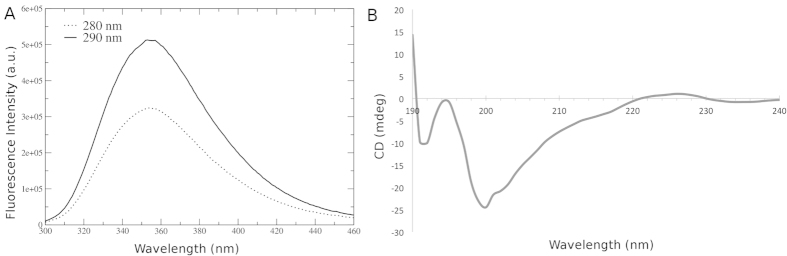
Experimental results of fusion peptide FP-1 in water at neutral pH and 300 K. (**A**) The fluorescence intensity at excitation of 280 and 290 nm; (**B**) Mean circular dichroism spectra obtained using 10 acquisitions.

**Figure 3:**
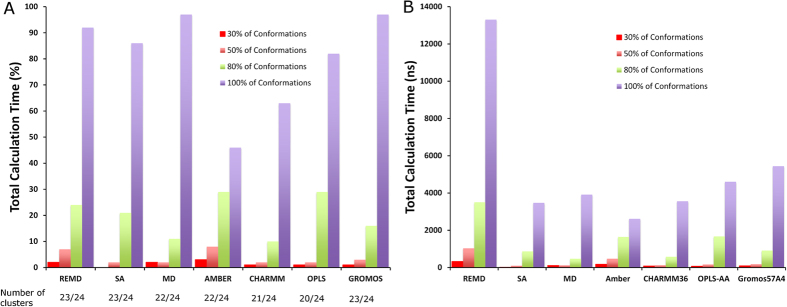
Results of the clustering analysis on the combined pseudo-trajectory of all methods and force fields for FP-1. This is shown (**A**) as a percentage of the total pseudo-trajectory length, and (**B**) as a function of total calculation time. Results are presented as the total required calculation time to explore 30, 50, 80 and 100% of all conformation clusters. The methods included four different force fields: Amber99SB*-ILDN-Q, CHARMM36, Gromos54A7 and OPLS-AA; as well as three methods: replica exchange molecular dynamics (REMD), simulated annealing (SA) and conventional molecular dynamics (MD). The result of each sampling method utilized a combined trajectory of all force fields, and likewise, the results for each force field involved a combined trajectory of all sampling methods.

**Figure 4:**
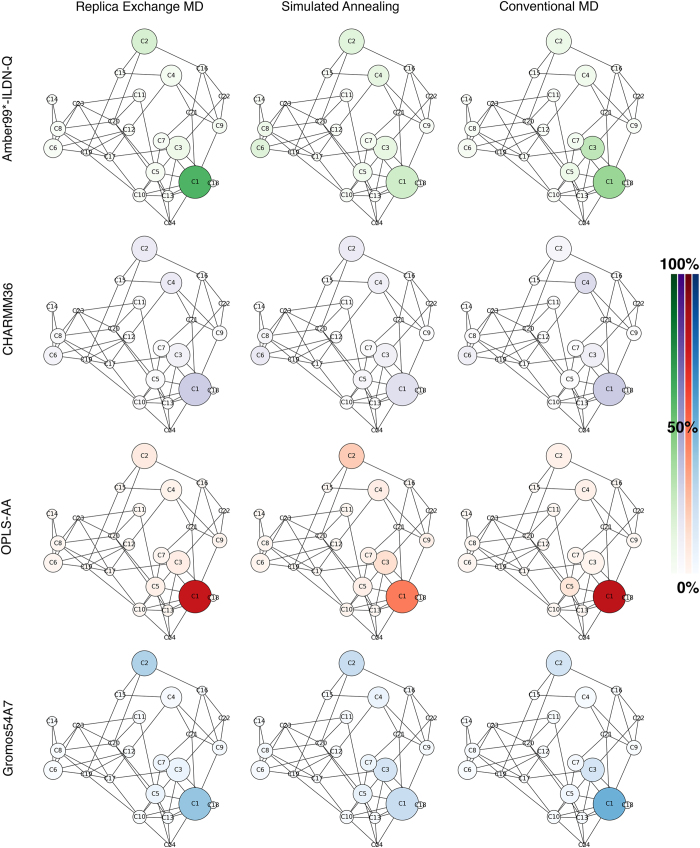
Network analysis of clusters explored by the pseudo trajectory composed of all methods (REMD, SA, and conventional MD) as well as four FFs (Amber99SB*-ILDN-Q, CHARMM36, OPLS-AA and Gromos54A7). Graph edges represent structural similarity (RMSD < 0.45 nm) of individual cluster centroid structures. The size of individual nodes is proportional to the overall population of a cluster in the entire combined trajectory of all methods and FFs. Colours indicate relative populations of individual clusters using a specific method and force field.

**Figure 5:**
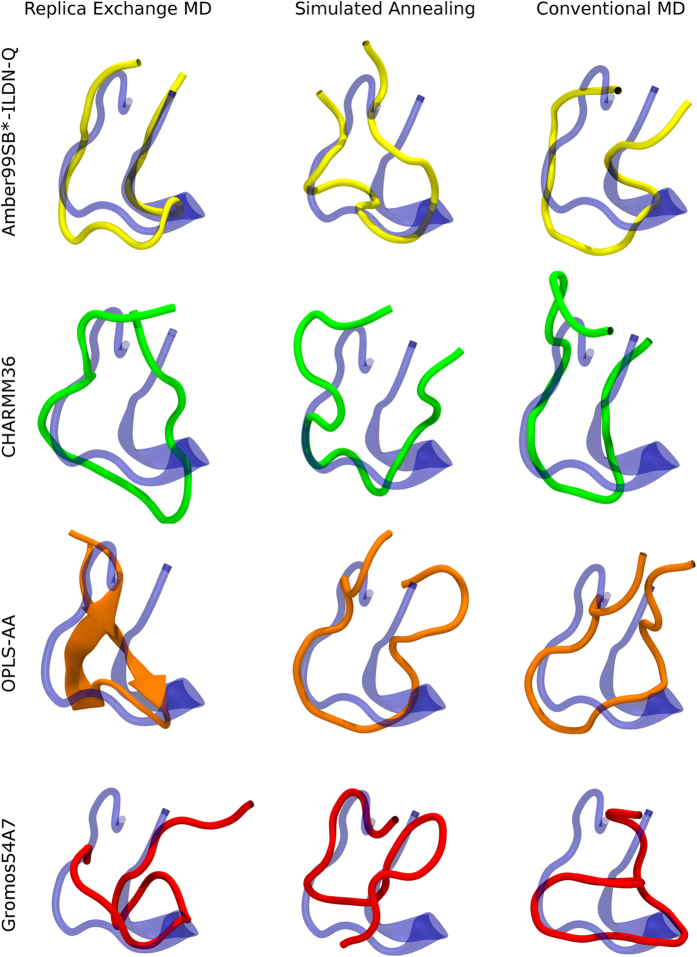
Comparison of structures generated by simulation and experiment. Representative snapshots are shown for the first cluster of FP-1 explored by REMD, SA and MD using various force fields. The protein is shown in cartoons representation, for: the crystal structure (PDB: 1OAN, blue), Amber FF (yellow), CHARMM36 FF (green), OPLS-AA (orange), and Gromos (red).

**Figure 6:**
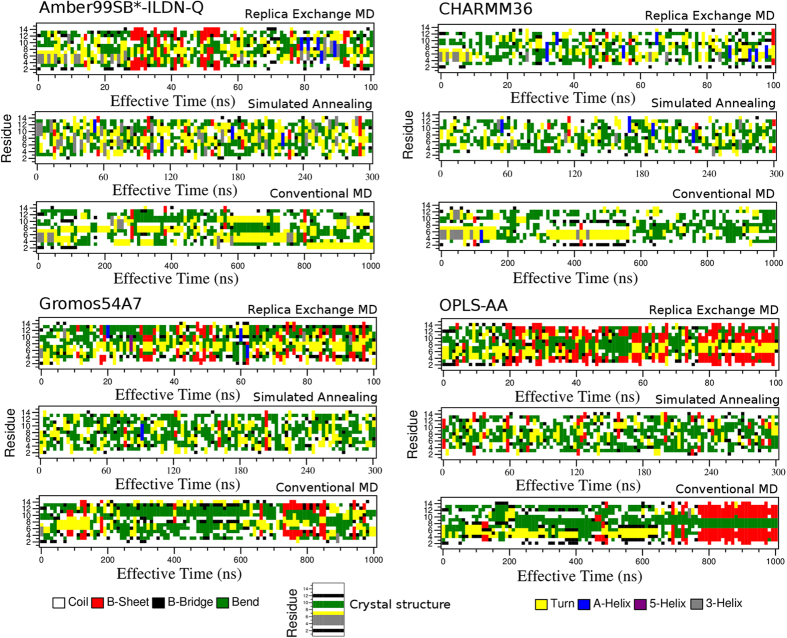
Secondary structure analysis of FP-1. Propensity for per-residue secondary structural features as a function of simulation time, shown for all combinations of the four FFs and three sampling methods. For reference, the structural features of the crystal structure are shown at the bottom.

Clustering analysis on independent trajectories of a given method and FF invariably yielded a primary cluster accounting for at least >30–50% of all configurations (Table 2). Hence, it was used in subsequent analysis including structural alignment with X-ray structures. The X-ray structure of FP is well conserved across different experimental conditions (Figs 1 and 5). Visual analysis indicates that using Amber and CHARMM36 with both REMD and MD yielded a well-aligned hairpin-like fold with a central turn, that is contrasted by a less well aligned structure generated with SA. The older CHARMM22/CMAP FF (Supplementary Fig. 18) yielded partially α-helical conformations irrespective of sampling methodology, whilst conformations generated with Gromos were random coil and deviated significantly from the experimental structure. Finally, OPLS-AA yielded significantly better crystallographically-aligned, hairpin-like structures, but showed a preference for β-sheet conformations for REMD and conventional MD (Fig. 6).

In order to quantitatively assess this, the Q_H_ as well as Cα RMSD with respect to the crystal structure was calculated for the most populated conformations (Table 2). In addition, each system was assessed in terms of time-dependent secondary structure (Fig. 6, Supplementary Fig. 19 for CHARMM22/CMAP). For a given FF, the highest structural alignment (Q_H_ values) was obtained using REMD, with the exception of CHARMM22/CMAP, in which case similar Q_H_ values of ~0.5 were obtained for REMD and conventional MD. In particular, using REMD, Amber, CHARMM36 and OPLS-AA provided the highest Q_H_ values compared to other combinations of method and FF, corresponding to values of 0.61, 0.52 and 0.66, respectively. Interestingly, SA yielded structures that were farthest in conformation from the crystal structure for any particular FF. Although this method may be useful for exploring conformational space, it therefore does not appear as applicable for efficiently sampling conformations which align well with the crystallographically-observed, restricted hairpin-like fold. When considering secondary structure propensity across FFs (Fig. 6, and Supplementary Fig. 19 for CHARMM22/CMAP), many peptide conformations uniformly spread over the simulation time were explored with REMD and SA, consistent with the multiple clusters sampled (Supplementary Figs 6–10). However, in the case of conventional MD, periodic shifts from one secondary structure state to another were observed. OPLS-AA in particular favoured a β-sheet/hairpin towards the end of the simulation from around 800 to 1,000 ns. Previously it was shown that the CHARMM22/CMAP tends to overestimate the occurrence of α-helical secondary structure^27^,^31^. We observed that all simulation methods yielded partially helical structures when using this FF (Supplementary Figs 18 and 19), which may explain the reduced number of representative clusters obtained (Table 2). In contrast, the improved CHARMM36 FF yielded better aligned, unstructured conformations (Figs 5 and 6), consistenent with its parameterization against a wide range of unstructured peptides^42^.

Interestingly, only CHARMM22/CMAP in combination with REMD reproduced the 3_10_-helix for residues W101, G102 and N103 observed in crystal structures (Supplementary Fig. 18), despite the fact that the peptide structure as a whole did not align well with the experimental structure (Q_H_ = 0.48, RMSD = 0.58 nm). Taking into account our findings from CD and spectrofluorometry experiments as well as previous observations for a shorter FP^18^, it appears that this region is biased towards a 3_10_-helix in the crystallographic environment. This would be consistent with the propensity for CHARMM22/CMAP to prefer helical structures due to fitting to X-ray data. In solution, the single hydrogen bond associated with the 3_10_-helix within the flexible FP may be too weak and/or entropically unfavored to compete with local solvent. In Supplementary Table 2 the mean secondary structure propensity within the most dominant cluster is presented. Consistent with the analysis above for the entire conformational ensemble, at least 50% of conformations in the dominant cluster for all combinations of method and FF are characterized by a turn. However, significant propensity for other secondary structural motifs in the dominant cluster was only observed for OPLS-AA, with as much as 60% β-sheet structure in the case of REMD (see also Fig. 5), and for CHARMM22 with a combination of α-helix and 3_10_-helix conformations observed across methods (see also Supplementary Fig. 18).

When comparing FFs with REMD, the RMSD between each dominant cluster and the crystal structure was significantly higher for the OPLS-AA FF in comparison with Amber or CHARMM36 (0.65 versus 0.22 and 0.34 nm respectively, Table 2). This is confirmed by the preference for β-sheet conformations in comparison with the states mostly containing undefined secondary structures yielded by Amber and CHARMM36 (Figs 5 and 6). Interestingly, the performance of OPLS-AA was significantly better when using conventional MD, where the dominant cluster RMSD was 0.36 nm. Similarly, REMD (RMSD = 0.34 nm) performed worse than MD (RMSD = 0.24 nm) for CHARMM36. In contrast, the Amber system performed slightly worse upon switching to conventional MD (RMSD = 0.27 nm). Although Amber with REMD yielded a dominant conformation that was closest to the crystal structure (highest Q_H_ and the lowest RMSD) in comparison with all other FF/method combinations, its application in combination with SA provided a poorly aligned structure, with a Q_H_ of 0.31 and RMSD of 0.56 nm. However, it should be noted that SA consistently yielded the least aligned conformations across all force fields. This likely arises from the sampling of transient higher energy states far from the global minimum associated with SA. Sampling using conventional MD yielded significantly better crystallographically-aligned backbone conformations than SA in all cases. Irrespective of sampling method, the Gromos FF did not seem to describe the FP fold well, as indicated by global Q_H_ values of ~0.3–0.4. This is supported by the RMSD values between representative clusters generated by pairs of FFs (Supplementary Table 1). RMSDs of ~0.2–0.5 nm were observed between Amber, OPLS-AA, CHARMM22/CMAP and CHARMM36, whereas all values for Gromos were ~0.6–0.8 nm, thus indicating that the sampled conformations were rather different from those generated by the remaining FFs.

#### Free Energy Landscapes

**Figure 7:**
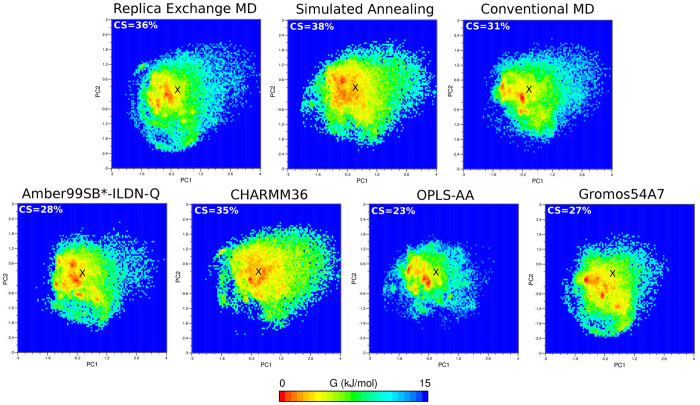
Energy landscape analysis for sets of FFs or sampling methods. Contour plots representing the free-energy landscapes of FP-1 as a function of the first (PC1) and second (PC2) principal components are shown, comparing each sampling method across all FFs (top row) or each FF across each sampling method (bottom row). The location of the crystal structure on each plot is denoted with a cross. Covered surface (CS) corresponds to the percentage of the total free energy landscape area occupied in at least one frame of the analysed simulation data. The principal components were extracted from the combined trajectory of all methods and force fields (FFs) and subsequently eigenvectors were projected using combined trajectories of specific method or FF.

Principal component analysis (PCA) was performed to characterize the conformational free energy landscape of each system along the most dominant principal components (PCs). To facilitate comparison of the landscapes associated with a particular sampling method or FF, a pseudo-trajectory composed of all trajectories studied here (indicated by effective time in Table 1) was generated. Subsequently PCA was performed to define the maximum accessible space defined by the two PCs associated with all methods and FFs. The first two components (PC1, PC2) of the entire combined trajectory captured approximately ~40% of the total variance, as shown in Supplementary Fig. 20. To compare the conformational dynamics generated with different sampling methods (REMD, SA, MD), the eigenvectors extracted from the pseudo-trajectory (all methods and FFs) were projected using all trajectory segments which involved a particular method (i.e. comprising of all FFs) onto the free energy surface. Likewise, eigenvectors from the pseudo-trajectory (all methods and FFs) were projected using a trajectory which involved a particular FF (i.e. comprising of all methods) in order to compare landscapes arising from each FF (Fig. 7). A similar approach was employed when comparing particular combinations of individual method and FF (Supplementary Fig. 21).

The percentage of the entire free energy landscape occupied at any point by the FP for each method was calculated to yield the “covered surface” (CS), as specified inset in Fig. 7. Out of all the sampling methods, SA yielded the largest CS (38%), compared to ~36% for REMD and ~31% for conventional MD, and sampled the greatest number of free energy minima (Fig. 7, Supplementary Fig. 21). However, the most populated conformations produced by SA did not reproduce the crystal structure conformation accurately, as shown above (Table 2, Fig. 5). Thus, SA samples many populated conformations distant from the X-ray structure as a result of the fast heating/cooling process and shows a tendency for system “runaway”^52^. Whilst all methods significantly sampled conformations around the experimental structure, and the crystal structure tended to coincide with a free energy minimum, the relative populations varied significantly (Fig. 7). For instance, REMD appeared to sample a few highly populated energetic minima which yielded better experimentally aligned structures (Table 2, Fig. 5). Although conventional MD explored the least number of energetic minima (Fig. 7, Supplementary Fig. 21), the most populated conformations also tended to be close to the X-ray structure. In terms of FF performance, the crystal structure was very close to the lowest energy state for all FFs. Furthermore, in comparison to other force fields, CHARMM36 explored the largest conformational space (Fig. 7, Supplementary Fig. 21) in agreement with the observation that it explored highest number of conformations of all FFs (Table 2).

### Comparative conformational dynamics of FP-1, FP-2, FP-3 and FP-4

**Figure 8:**
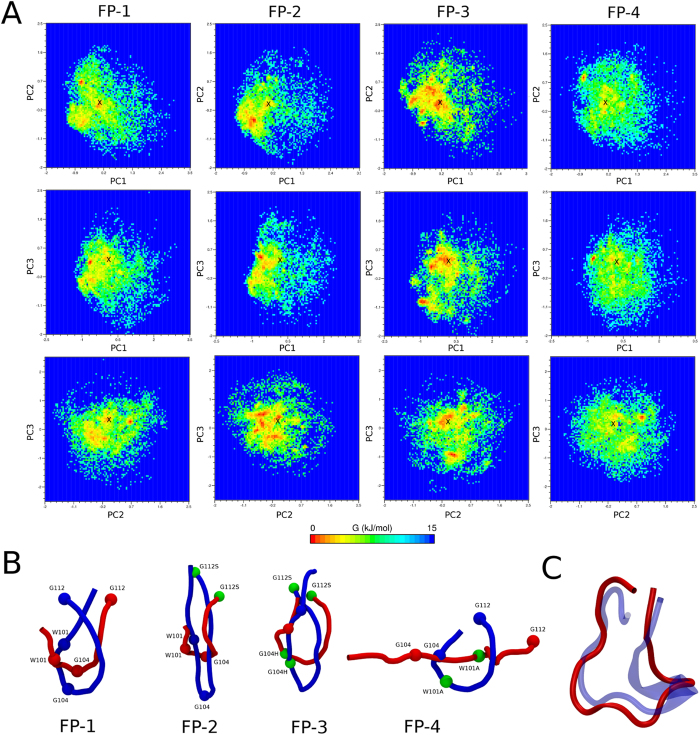
Conformational characterization of all FP variants. (**A**) Energy landscape analysis for all fusion peptide sequences. Contour plots representing the free-energy landscapes of FP-1, FP-2, FP-3, FP-4 as a function of the first and/or second and/or third PCs are shown for each FP system, using REMD/Amber99SB*-ILDN-Q. The location of the crystal structure on each plot is denoted with a cross; (**B**) Dominant FP motions. The extreme conformations (blue and red) along the first principal component (PC1) of all fusion peptides (labelled) are shown. The Cα atoms of mutated residues between FP variants are shown as green spheres; (**C**) Alignment of simulated FP-4 and experimental FP-1 peptides. A representative snapshot of the most populated cluster of FP-4 (red cartoons representation) generated by REMD with the Amber FF is shown, aligned to the FP-1 crystal structure (PDB:1OAN, blue cartoons representation).

Since the most populated conformations of FP-1 when using Amber best aligned with the crystal structure (Fig. 5, Table 2), we used the same FF to compare the dynamics of two wild-type variants (Table 1), FP-2 and FP-3, using REMD, SA, and MD. In addition, REMD was applied in combination with Amber to the non-fusogenic mutant sequence, FP-4. A summary of the accuracy of the most populated cluster arising for each is presented in Supplementary Table 3; accompanying simulation snapshots and free energy landscapes for FP-2 and FP-3 are shown in Supplementary Figs 22 and 23. Similarly to FP-1, REMD provided the highest Q_H_ values compared to SA and MD for both remaining wild-type FP variants, though RMSDs were ~0.1 nm lower than for FP-1 and transient secondary structural regions were also observed (Supplementary Fig. 22). Although SA again provided the highest number of representative structures (Supplementary Table 3) and broadest free energy landscape (Supplementary Fig. 23), its most populated cluster aligned poorly with the crystal structure, as also observed for FP-1. Like FP-1, calculation of the mean secondary structural propensity across the most populated cluster across the other FP sequences (Supplementary Table 4) revealed a dominance of turns (at least ~80%) and a lack of helical motifs, irrespective of sampling method. Surprisingly, the best aligned structure across all method/FF combinations and peptide sequences corresponded to FP-4 using REMD with Amber. This was observed despitethe lack of the key W101 residue, which has been suggested to be an important stabilizing determinant of the FP tip structure, forming a hydrophobic cluster with F108^12^,^15^,^16^,^17^, and under some circumstances, L107^12^. Thus, it yielded a Q_H_ of 0.71 and the lowest of all RMSDs (0.19 nm). The most populated FP-4 simulation conformation aligned with the wild-type FP-1 crystal structure is presented in Fig. 8C.

Our simulations suggest that the single-point mutant peptide FP-4, experimentally shown to completely abolish the membrane fusion process^12^ as well as inhibit Dengue primary and secondary infection^21^, likely possesses the same equilibrium solvated structure as FP-1, and hence all fusion peptides belonging to flavivirus family (Fig. 1). However, it is possible that FP-4 (and indeed all FP variants) may exhibit different functionally relevant dynamics. To explore this hypothesis, we performed PCA and extracted eigenvectors for a combined pseudo-trajectory generated for all FP construct trajectories using REMD with Amber99SB*-ILDN-Q (4 × 3600 ns). Due to the fact that the variance of PC1 captured only 19% of all principal components and PC2 just over 13%, we analysed PC3, which also accounts for nearly 13% (Supplementary Fig. 24). Subsequently, the PC1/PC2, PC1/PC3 and PC2/PC3 combinations were projected onto a free energy surface for each FP trajectory (Fig. 8A). In addition, in order to visualize the most dominant motions of each fusion peptide, the extreme conformations along PC1 are presented in Fig. 8B.

Although very similar to FP-1, the free energy landscapes (Fig. 8A) for FP-2 and FP-3 reveal some additional well-defined neighbouring free energy minima further from the X-ray structure. In Fig. 8B it may be observed that the dominant component of the FP-2 and FP-3 motion along the first eigenvector is associated with a reversible “elongation” of the FP. Similarly, the FP-1 extreme conformations correspond to an opening-closing motion leading to more compact states. Although similar in motion to FP-1, the most dominant component of FP-4 exhibited much wider and more flexible extremes. It may be observed from the PCA analysis that the FP-4 (W101A) peptide explores a significantly wider free energy landscape for all combinations of components represented in comparison to the remaining flavivirus family FPs, with fewer defined minima, and hence more flexibility (Fig. 8B). By using the previously validated protocol combining REMD and Amber, hairpin-like conformations with a central turn were observed within the first cluster which covered 63, 76, 67 and 60% of explored conformations for FP-1, FP-2, FP-3 and FP-4 respectively (Supplementary Fig. 25). It may be observed that, similarly to the crystal structure, the hydrophobic residues W101 and F108 pack closely together, whilst adopting variable orientations in the absence of crystal contacts or a membrane environment. Hence, we hypothesise that the hydrophobic triad (W101 or A101, L107 and F108) naturally biases the FP to pre-formation of hairpin-like structures, and that the loss of W101 destabilizes the “hydrophobic core” resulting in greater flexibility and hence a tendency to explore non-hairpin FP conformations. Thus, in addition to an overall loss of lipophilicity, the W101A mutation likely results in sampling of functionally non-productive structures – both would be expected to reduce the propensity for membrane interaction and/or its fusogenic capacity, as observed via fluorescence spectroscopy^12^.

## Discussion

In this study, we have combined extensive MD simulation sampling with spectroscopic characterization to assess the capacity for different combinations of sampling approach and FF to accurately and efficiently predict the structure and dynamics of unstructured peptides as represented by variants of the flavivirus fusion peptide. We first confirmed that convergence had been achieved for all systems from our extensive sampling amounting to ~40 μs. Consistent with this, we showed that all methods sampled approximately the same total number of peptide conformations. Somewhat surprisingly, whilst most such conformations were explored rapidly within <10% of the computational time across all methods, conventional MD sampled all possible conformations within significantly shorter times than REMD or SA. Nevertheless, the enhanced sampling approaches ensured that the most populated configurations remained more persistent, compared to MD which yielded conformations trapped within different local minima over hundreds of nanoseconds, despite the unstructured and relatively flexible nature of the fusion peptide.

While all methods sampled conformations close to the X-ray structure, the corresponding energetic minima sampled via REMD tended to be significantly more populated than other methods. This was particularly the case in combination with the Amber99SB*-ILDN-Q forcefield, in particular for FP-1 and FP-4 where the highest alignment was reached. Nevertheless, the combination of REMD and Amber suggests that this protocol may be most appropriate for accurately predicting, *ab initio*, the structure of small unstructured peptides. Indeed, the predictive accuracy appears to be sensitive even to the local environment: not only were the most populated FP-1 clusters obtained by this approach close to the X-ray structure, but the dominant simulated conformations lacked a short, central 3_10_-helix present in the crystallographic state but absent in the presence of detergent^12^, and as shown here using CD and spectrofluorometry, in solution. CHARMM36 also yielded a well aligned conformation, conserving the random coil structure. The use of other FFs led to predicted conformations that deviated more from the X-ray structure. In some cases conformations exhibited biases towards particular secondary structural types, including partially α-helical (CHARMM22/CMAP) or β-sheet conformations (OPLS-AA), On the other hand, the convergence properties of each FF should also be borne in mind. Simulation using Gromos and OPLS tended to take almost twice as long as Amber and CHARMM36 to sample all accessible conformations. Thus, the use of particular FFs may be more desirable to simulate unstructured peptides, for example, when attempting to rapidly sample rare conformations in the phase space. Finally, whilst Gromos54A7 yielded random coil conformations that were substantially different from those sampled by all other FFs, it should be noted that a cutoff-truncated electrostatic interaction with reaction field correction is typically used with the GROMOS FFs. For consistency, the electrostatics were treated using the Particle Mesh Ewald (PME) approach in all systems here, and hence, we cannot rule out that different conformational ensembles might have otherwise been obtained.

From a therapeutic perspective, the high apparent degree of conservation in both sequence and structure of FPs across the flaviviruses makes them a promising target for antiviral drugs and antibodies^12^,^13^. For example, the potential for FPs from Dengue to act as an epitope and/or inhibitory site is already supported by a range of structural^20^, mutational^21^, and biochemical^53^,^19^ data. In this study, we were able to show that the equilibrium structure of the W101A FP mutant is remarkably similar to those of the wild-type FP variants, but that it samples far more open, unfolded conformations. As well as helping to explain how the W101A mutation disrupts fusogenic activity, this makes clear that even a short peptide sequence exhibits significant thermal fluctuations that may be probed in the context of rational design of therapeutics. This is further emphasized by our computational and experimental confirmation that a 3_10_-helix within a known antibody epitope^20^,^21^ may be lost under non-crystallographic conditions, or when the remainder of the E protein or entire viral envelope are missing. By defining a reliable combination of sampling protocol and FF, we are now in a position to explore the conformational landscape of flavivirus FPs and related peptide fragments. These landscapes may, for example, be utilized to further characterize some of the well populated but non-crystallographic minima observed here to further expand virtual screening efforts. Moreover, rare conformations common to several serotypes may be sought in order to design restricted/cross-linked peptides that may subsequently be tested for generation of flavivirus polyclonal antibodies^19^. Given the ongoing problems of infectious disease and death associated worldwide with the flavivirus family^1^, such approaches may thus help to fulfil the urgent need for effective drugs and vaccines^5^,^6^.

## Methods

### Structural alignment

The dominant conformation was extracted via clustering analysis performed on the total trajectory of a given combination of method and FF. Structural alignment of FP conformations from the PDB as well as representative conformations obtained from simulation was performed using STAMP structural alignment within the MultiSeq module of VMD^54^. Q_H_ measures the structural identity between a reference structure and structure of interest, and represents a normalized fraction of native contacts between aligned residues in two proteins, after taking into account gaps and insertions, summed over all pairs of Cα atoms (excluding nearest neighbours)^55^. Q_H_ ranges between 0 (completely non-matching structures) and 1 (identical structures). A value of 1 corresponds to identical structural alignment while 0 to ~0.3 represents poor structural alignment. In addition, the RMSD was calculated between the crystal structure and structure of interest for Cα atoms of the FPs, following pairwise least-squares fitting. A summary of available crystal structures of the FP-1, FP-2 and FP-3 is available in Supplementary Data 1.

An alignment of all available FP crystal structures for each of the three fusion peptides studied is presented in Fig. 1. Key aromatic residues are indicated in a stick representation^13^. Near identical structures were apparent for crystal structures of all FPs from the flavivirus family. In Supplementary Tables 5, 6 and 7, the pair-wise RMSDs as well as Q_H_ factor values between all pairs of crystal structures are presented. For FP-1, the maximum RMSD and Q_H_ pairwise values corresponded to 0.05 nm and 0.93 respectively (Supplementary Table 5). For FP-2, the maximum pairwise RMSD is 0.08 nm and Q_H_ is 0.90 (Supplementary Table 6). In both cases, these values correspond to highly aligned structures. For FP-3 with two available crystal structures obtained under different conditions, the RMSD was 0.02 nm and Q_H_ was 0.99 (Supplementary Table 7), showing excellent alignment, suggesting that the equilibrium FP-3 conformation is effectively unchanged by pH. Hence, further analysis of the most persistent structures obtained from MD simulations were conducted in comparison with single X-ray structures for FP-1 (PDB: 1OAN^56^), FP-2 (PDB: 1UZG^57^), and FP-3 (PDB: 1URZ^58^). The maximum standard deviations of RMSD and Q_H_ between crystal structures (Supplementary Table 5–7) served as an error threshold when comparing alignments from simulations with the crystal structures. As no experimental structure is available for FP-4, the MD-generated conformations were aligned to the crystal structure used for alignment of FP-1 (PDB: 1OAN) and the highest observed error across experimental structures was utilized (i.e. RMSD of ± 0.08 nm and Q_H_ of ± 0.10). It may also be observed (Fig. 1) that almost all aromatic side chains aligned extremely well. The sole exception is FP-2 (PDB: 3G7T^59^, blue wireframe format in Fig. 1) residue W108, which for unknown reason^59^ orients its side chain in the opposite direction compared to the other FP X-ray structures.

### Circular dichroism and spectrofluorometry

We employed CD and spectrofluorometry in order to assess the secondary structure of FP-1 (15 residues) in water. The FP-1 sequence was synthesized by Biomatik Co. (USA) with >95% purity measured by high performance liquid chromatography (HPLC). The sample for all measurements consisted of 1 mg of the peptide dissolved in 1 ml of PBS buffer. All measurements were conducted at 300 K, a pressure of 1 bar and neutral pH, corresponding to conditions used in all our MD simulations (Table 1). For CD measurements a Chirascan^TM^ – plus CD spectrometer (Applied Photophysics Ltd, UK) was used. For spectrofluorometry measurements, a Model 814 PMT Housing (Photon Technology International, USA) was employed. The CD result was obtained from multiple acquisitions of 10 spectra.

### Simulation system setup

The crystal structure of FP-1 corresponded to the Dengue virus serotype 2 (DENV-2) ectodomain E protein (protein data bank entry: 1OAN^56^, residues 98–112): NH_3_^+^-DRGWGNGCGLFGKGG-COO^–^ obtained as a dimeric conformation (mature virus) under conditions of neutral pH. All *in silico* point mutations to generate FP-2, FP-3 and FP-4 were constructed based on this input structure using PyMol (https://www.pymol.org). Each FP construct was placed in the centre of a cubic box (4.5 × 4.5 × 4.5 nm) and solvated with approximately 3,000 TIP3P^60^ water molecules using Amber99SB*-ILDN-Q and CHARMM 22/CMAP. In the case of Gromos54A7, the SPC^61^ water model was used while OPLS-AA employed TIP4P^60^. All ionizable residues in FP-1, FP-2, and FP-4 (Table 1) along with the termini were treated in their fully charged state, yielding an overall charge of +1. This is equivalent both to conditions of neutral pH (as found in the spectroscopy experiments for FP-1 described here) and to endosomal conditions (pH 5.5). In the case of FP-3, the histidine was treated in its protonated form (i.e. equivalent to endosomal conditions), resulting in an overall charge of +2. It should be noted that alignment of known crystal structures corresponding to FP-3 (Table 1) from: i) the mature virus (neutral pH, PDB: 1SVB) and ii) the post-fusion conformation (acidic pH, PDB: 1URZ) revealed nearly identical structures. In order to neutralize the overall charge of each system, one or two chloride ions were added. All MD simulations were performed with the GROMACS 5.0.2 package^62^. Equations of motion were integrated through the Verlet leapfrog algorithm with a 2 fs time step. Bond lengths were constrained with the LINCS algorithm^63^. The cutoff distance was 1.4 nm for the short-range neighbour list and van der Waals interactions. The Particle Mesh Ewald (PME) method was applied for the long-range electrostatic interactions with a 1.4 nm real space cutoff^64^. The velocity rescale thermostat with an additional stochastic term^65^ and Berendsen barostat^66^ were used to maintain the temperature and pressure (1 bar) in all simulations. Initial velocities were set according to the Maxwell distribution. Initial configurations were minimized using the Steepest Descent algorithm. Periodic boundaries were applied in all directions. All simulations were performed on an in-house Linux cluster of 7 nodes of 2 GPUs (Nvidia K20) and 20 CPUs (Intel® Xeon® CPU E5-2680 v2 @ 2.8 GHz) each.

### Replica exchange molecular dynamics (REMD) simulations

REMD was used to study the dynamics of all FP constructs. Application of each force field employed 36 replicas with temperatures ranging from 297, 300, 303.47, …, 427.51 K (3 to 4 K increments) generated by the temperature predictor for parallel tempering simulations^67^. The exchange probability corresponded to 0.2. Each system replica prior to the production run was equilibrated using 1 ns NVT followed by 1 ns NPT ensembles with positions of the heavy atoms restrained. Coordinates between replicas were exchanged every 2 ps and the production run (NPT) corresponded to 100 ns at a given temperature replica. Thus, each REMD run amounted to 3,600 ns of total simulation time. All analyses of REMD corresponded to the 100 ns trajectory at 300 K (effective, Table 1). Examples of the potential energy distributions along the temperature increments reveal excellent overlap (Supplementary Fig. 26).

### Simulated annealing (SA)

The equilibrated conformations at 300 K were used as input for SA. In the SA production runs (NPT ensembles) each system was driven over 100 cycles of 10 ns (1,000 ns of simulation time). Each 10 ns cycle corresponded to: 1 ns of heating up from 300 K to 450 K; 4 ns at 450 K; 2 ns of cooling down to 300 K; 3 ns at 300 K. All analyses were performed for the last 3 ns of each cycle (300 K), totalling 300 ns (effective time).

### Conventional MD simulations

Simultaneously, all equilibrated systems were subject to conventional MD in the NPT ensemble at 300 K for 1,000 ns each.

### Simulation analysis

In order to examine the accuracy of each method and force field, the time-dependent structural properties of the peptides were assessed. Secondary structure was analysed using DSSP. Pair-wise clustering was performed using the Gromos method^24^ for the Cα atoms. In this method the number of neighbours for each conformation using the RMSD cut-off is counted. The structure with largest number of neighbours is considered as the centre of the cluster forming a cluster together with its neighbours. In this manner, conformations belonging to the cluster are eliminated from the pool until it becomes empty. Thus a series of non-overlapping clusters are obtained. For consistency in clustering, a constant RMSD cut-off of 0.45 nm was used for all the systems. In convergence analysis, in order to compare MD simulations results with experimental values, the radius of gyration, secondary structure, the solvent accessible surface area (SASA) as well as end-to-end distance corresponded to the crystal structure (PDB: 1OAN).

For time dependent convergence analysis the pseudo-trajectory involving three methods (REMD, SA and MD) as well as four FFs (Amber99SB*-ILDN-Q, Charmm36, OPLS-AA, Gromos54A7) were concatenated and subsequently clustered (as described above) yielding 24 distinct clusters. Subsequently, pairwise RMSDs were calculated for the 24 cluster centroid structures. The resulting pairwise distance matrix was used to construct a graph illustrating the structural relations of the different clusters. Edges corresponding to an RMSD of <0.45 nm were pruned. Cluster populations were encoded as node sizes. The populations of each cluster using a specific method or FF were encoded as the node colour.

Covariance analysis and PCA^68^ was conducted for Cα atoms using tools within the GROMACS package. In order to illustrate the sampled conformational space and folding/unfolding events, the free-energy landscapes for each system were constructed along the first (PC1) and the second (PC2) components, using: 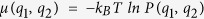, where 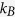 is the Boltzmann constant, 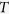 is absolute temperature, and 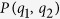 is the probability density function for 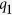 and 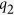, the corresponding principal components. In order to compare free energy landscapes of each method and/or force field, the covariance matrix as well as eigenvectors were extracted from the combined trajectory of all methods and force fields. Subsequently, the PC1 versus PC2 components were projected as free energy landscapes for each system/method. The covered surface (CS) of the free energy landscape corresponded to the percentage of occupied bins in the PC1/PC2 matrix.

## Additional Information

**How to cite this article**: Marzinek, J. K. *et al.* Characterizing the Conformational Landscape of Flavivirus Fusion Peptides via Simulation and Experiment. *Sci. Rep.* **6**, 19160; doi: 10.1038/srep19160 (2016).

## Supplementary Material

Supplementary Information

## Figures and Tables

**Table 1:** Summary of FP sequences studied together with employed methodology and calculation/effective time.

Fusion Peptide	Sequence	Corresponding Viruses	Employed Methods	Force Field Employed	Calculation time (ns)	Effective Time at 300 K (ns)
FP-1	DRGWGNGCGLFGKGG	DENV-2	REMD	Amber99SB*-ILDN-Q	36 × 100	100
		DENV-4		CHARMM36	36 × 100	100
				Gromos54A7	36 × 100	100
				OPLS-AA	36 × 100	100
				CHARMM22/CMAP	36 × 100	100
			SA	Amber99SB*-ILDN-Q	1000	300
				CHARMM36	1000	300
				Gromos54A7	1000	300
				OPLS-AA	1000	300
				CHARMM22/CMAP	1000	300
			MD	Amber99SB*-ILDN-Q	1000	1000
				CHARMM36	1000	1000
				Gromos54A7	1000	1000
				OPLS-AA	1000	1000
				CHARMM22/CMAP	1000	1000
FP-2	DRGWGNGCGLFGKGS	DENV-1, DENV-3	REMD	Amber99SB*-ILDN-Q	36 × 100	100
		WNV, JEV, YFV, SLEV	SA	Amber99SB*-ILDN-Q	1000	300
			MD	Amber99SB*-ILDN-Q	1000	1000
FP-3	DRGWGNHCGLFGKGS	TBE	REMD	Amber99SB*-ILDN-Q	36 × 100	100
			SA	Amber99SB*-ILDN-Q	1000	300
			MD	Amber99SB*-ILDN-Q	1000	1000
FP-4	DRGAGNGCGLFGKGG		REMD	Amber99SB*-ILDN-Q	36 × 100	100

Calculation time corresponds to the total sampling time required for a given method, and the effective time to the length of the resulting analysed trajectory. The employed force fields include Amber99SB*-ILDN-Q^41^, CHARMM22/CMAP^45^,^46^, CHARMM36^42^, Gromos54A7^43^, and OPLS-AA^44^. The methods involved: replica exchange molecular dynamics (REMD), simulated annealing (SA), and conventional molecular dynamics (MD).

**Table 2:** Assessment of most populated cluster for the FP-1 peptide using various methods and force fields.

Method	Force Field	No. of clusters obtained	% of the total effective simulation time explored by the most populated cluster	Comparison of crystal structure vs the most populated cluster, Q_H_^a^	Comparison of crystal structure vs the most populated cluster, RMSD (nm)^b^
REMD	Amber	16	63	0.61	0.22
	CHARMM36	19	38	0.52	0.34
	OPLS-AA	13	72	0.66	0.65
	Gromos	18	55	0.30	0.88
	CHARMM22	11	63	0.48	0.58
SA	Amber	19	58	0.31	0.56
	CHARMM36	19	33	0.37	0.49
	OPLS-AA	17	50	0.39	0.49
	Gromos	21	33	0.33	0.69
	CHARMM22	12	60	0.31	0.52
MD	Amber	18	46	0.50	0.27
	CHARMM36	18	48	0.46	0.24
	OPLS-AA	8	83	0.37	0.36
	Gromos	16	68	0.37	0.46
	CHARMM22	7	88	0.49	1.04

Q_H_ refers to the structural identity between the central structure of the most populated cluster and crystal structure PDB 1OAN. The RMSD was measured between Cα atoms of this conformation and the crystal structure.

^a^The maximum pairwise deviation in Q_H_ values between all aligned FP-1 crystal structures was 0.07.

^b^The maximum pairwise deviation in RMSD between all aligned FP-1 crystal structures was 0.05 nm.
