# Supplementary material for: Characterizing the Conformational Landscape of Flavivirus Fusion Peptides via Simulation and Experiment
Source: Sci Rep. 2016 Jan 20;6:19160. doi: 10.1038/srep19160 (PMC4726195; doi:10.1038/srep19160)
Supplement: Supplementary Information [file srep19160-s1.pdf]

Characterizing the Conformational Landscape of Flavivirus Fusion Peptides via Simulation and Experiment – SUPPORTING INFORMATION

Jan K Marzinek[1, 2], Rajamani Lakshminarayanan[3], Eunice Goh[3], Roland G. Huber[2], Sadhana Panzade[2], Chandra Verma[1, 2, 4,*], Peter J. Bond[1, 2,*]

[1] National University of Singapore, Department of Biological Sciences, 14 Science Drive 4, Singapore 117543
[2] Bioinformatics Institute (A*STAR), 30 Biopolis Str., #07-01 Matrix, Singapore 138671
[3] School of Biological Sciences, Nanyang Technological University, 60 Nanyang Drive, Singapore 63755
[4] Singapore Eye Research Institute, The Academia, 20 College Road, Discovery Tower Level 12, Singapore 169856

[*] Corresponding authors:

Dr Peter J. Bond
Bioinformatics Institute (A*STAR)
30 Biopolis Str.
#07-01 Matrix
Singapore 138671
Email: peterjb@bii.a-star.edu.sg

Dr Chandra Verma
Bioinformatics Institute (A*STAR)
30 Biopolis Str.
#07-01 Matrix
Singapore 138671
Email: chandra@bii.a-star.edu.sg

Supplementary Data 1. A summary of available crystal structures used for structural alignment.

X-ray structures of the dimeric E protein conformation (corresponding to the mature virus) from DENV-2 revealed a hydrophobic ligand binding pocket exposing FP-1 (D^{98}RGWGNGCGLFGKGG^{112}) which promotes the fusion process (PDB entries 1OKE and 1OAN[1] solved in the presence and absence of n-octyl-β-D-glucoside respectively). The main difference between those structures lies in residues 268-280 with the FP region unaffected, hence only one structure (PDB: 1OAN) was used in our study. In addition, FP-1 has been resolved by X-ray diffraction as a part of the DENV-2 trimeric (post-fusion) conformation (PDB entries 1OK8[2] and 1TG8[3]). The X-ray structure of DENV-2 dengue virus prM protein linked to glycoprotein E has also been solved, and fits well into the cryo-EM density maps of the entire immature virus at acidic and neutral pH (PDBs 3C6E and 3C5X[4] respectively) in which FP structure was not affected by the pH change. Thus, one structure (PDB: 3C5X) was considered in this study. Finally, the crystal structure of DENV-4 E glycoprotein ectodomain in complex with an Fab fragment of the chimpanzee monoclonal antibody 5H2 has been solved (PDB 3UAJ[5]). FP-2 (D^{98}RGWGNGCGLFGKGS^{112}) crystallographic structures are also available for several of these family members. The DENV-1 FP crystal structure was solved in the trimeric (post-fusion) conformation (PDB 3G7T[6]), as well as in DENV-3 in the mature virus (PDB 1UZG[7]). Furthermore, the WNV envelope proteins incorporating FP have been crystallized as a soluble monomeric unit similar to immature flaviviruses (PDB 2HG0[8]), in a conformation which resembles post-fusion conformations of other flaviviruses (PDB 2I69[9]), and in the mature conformation (PDB 3I50[10]). Moreover, the E protein containing this motif from JEV/SLEV has been crystallized (PDB 4FG0[11]). Finally, FP-3 (D^{98}RGWGNHCGLFGKGS^{112}) crystal structures represent the mature virus form (PDBs 1SVB[12]), as well as its post-fusion state (PDB 1URZ[13]).

Supplementary Table 1. Pair-wise RMSD (nm) matrix between representative structures obtained by cluster analysis for combinations of different method and force fields (FF). Methods involved include replica exchange molecular dynamics (REMD, simulated annealing (SA) and conventional molecular dynamics (MD) as well as four different force fields: Amber99SB*-ILDN-Q, Charmm36, OPLS-AA, Gromos54A7 and Charmm22/CMAP.

		REMD					SA					MD				
	**FF**	**Amber**	**Charmm36**	**OPLS-AA**	**Gromos**	**Charmm22**	**Amber**	**Charmm36**	**OPLS-AA**	**Gromos**	**Charmm22**	**Amber**	**Charmm36**	**OPLS-AA**	**Gromos**	**Charmm22**
**REMD**	**Amber**	0	0.28	0.33	0.63	0.42	0.35	0.43	0.38	0.62	0.38	0.17	0.35	0.26	0.65	0.32
	**Charmm36**	0.28	0	0.37	0.74	0.46	0.37	0.33	0.33	0.68	0.44	0.33	0.33	0.34	0.61	0.32
	**OPLS-AA**	0.33	0.37	0	0.62	0.51	0.32	0.48	0.41	0.57	0.34	0.36	0.45	0.33	0.57	0.40
	**Gromos**	0.63	0.74	0.62	0	0.67	0.66	0.68	0.62	0.37	0.59	0.47	0.77	0.51	0.51	0.50
	**Charmm22**	0.42	0.46	0.51	0.67	0	0.39	0.32	0.49	0.57	0.38	0.40	0.42	0.44	0.64	0.31
**SA**	**Amber**	0.35	0.37	0.32	0.66	0.39	0	0.39	0.37	0.56	0.31	0.34	0.44	0.36	0.59	0.30
	**Charmm36**	0.43	0.33	0.48	0.68	0.36	0.39	0	0.40	0.70	0.42	0.41	0.43	0.43	0.62	0.34
	**OPLS-AA**	0.38	0.33	0.41	0.62	0.49	0.37	0.40	0	0.58	0.44	0.37	0.46	0.32	0.62	0.38
	**Gromos**	0.62	0.68	0.57	0.37	0.57	0.56	0.70	0.58	0	0.54	0.52	0.73	0.44	0.50	0.37
	**Charmm22**	0.38	0.44	0.34	0.59	0.38	0.31	0.42	0.44	0.54	0	0.35	0.	0.37	0.63	0.38
**MD**	**Amber**	0.17	0.33	0.36	0.47	0.40	0.34	0.41	0.37	0.52	0.35	0	0.39	0.25	0.67	0.32
	**Charmm36**	0.35	0.33	0.45	0.77	0.42	0.44	0.43	0.46	0.73	0.	0.39	0	0.45	0.65	0.34
	**OPLS-AA**	0.26	0.34	0.33	0.51	0.44	0.36	0.62	0.32	0.44	0.37	0.25	0.45	0	0.62	0.36

Supplementary Table 2. Mean secondary structure propensity (%) across all members of the most dominant for FP-1. This was assessed for five different force fields (Amber99SB*-ILDN-Q, CHARMM36, OPLS-AA, Gromos54A7 and CHARMM22/CMAP) as well as three different methods (REMD, SA, and MD).

		**REMD**	**SA**	**MD**
**AMBER99SB*-ILDN-Q**	β-sheet	21	9	4
	α-helix	5	3	0
	Turn	80	81	90
	3_{10}-helix	17	26	12
**CHARMM36**	β-sheet	4	4	2
	α-helix	8	4	1
	Turn	76	52	74
	3_{10}-helix	9	7	10
**OPLS-AA**	β-sheet	60	17	33
	α-helix	0	0	0
	Turn	67	49	56
	3_{10}-helix	0	0	1
**GROMOS54A7**	β-sheet	28	4	19
	α-helix	3	1	0
	Turn	80	68	64
	3_{10}-helix	4	0	2
**CHARMM22**	β-sheet	0	1	0
	α-helix	34	15	20
	Turn	90	82	90
	3_{10}-helix	35	12	51

Supplementary Table 3. Assessment of the most populated cluster for FP-2, FP-3 and FP-4 using various methods in combination with the Amber99SB*-ILDN-Q force field. Q_H refers to the structural identity between the most populated cluster and the crystal structure (FP-1 and FP-4 – PDB: 1OAN; FP-2 – PDB: 1UZG; FP-3 – PDB: 1URZ). The RMSD was measured between Cα atoms of the most populated conformation and the crystal structure.
The Q_H and RMSD error bars correspond to the maximum pair-wise deviation of each FP crystal structure alignment.

**Fusion Peptide**	**Sequence**	**Method**	**No. of clusters Obtained**	**% of the total effective simulation time explored by the most populated cluster**	**Comparison of crystal structure vs the most populated cluster, Q_H [a]**	**Comparison of crystal structure vs the most populated cluster, RMSD (nm) [b]**
**FP-2**	DRGWGNGCGLFGKGS̲	REMD	15	76	0.54	0.34
		SA	19	46	0.34	0.71
		MD	18	52	0.48	0.51
**FP-3**	DRGWGNH̲CGLFGKGS̲	REMD	16	67	0.53	0.29
		SA	21	50	0.34	0.41
		MD	11	80	0.37	0.64
**FP-4**	DRGA̲GNGCGLFGKGG	REMD	19	60	0.71	0.19

[a] The maximum pairwise deviation in Q_H values between aligned crystal structures for FP-2, FP-3, and FP-4 was 0.10, 0.01, and 0.10, respectively.

[b] The maximum pairwise deviation in RMSD between aligned crystal structures was for FP-2, FP-3, and FP-4 was 0.08 nm, 0.02 nm, and 0.08 nm, respectively.

Supplementary Table 4. Mean secondary structure propensity (%) across all members of the most dominant cluster. This was assessed for FP-2 and FP-3 using Amber99SB*-ILDN-Q with three different sampling methods (REMD, SA and MD), and for FP-4 using REMD.

		**REMD**	**SA**	**MD**
**FP-2**	β-sheet	41	11	10
	α-helix	4	6	2
	Turn	78	83	87
	3_{10}-helix	22	19	20
**FP-3**	β-sheet	22	5	3
	α-helix	4	9	39
	Turn	80	79	88
	3_{10}-helix	21	12	27
**FP-4**	β-sheet	11		
	α-helix	4	-	-
	Turn	96		
	3_{10}-helix	19		

Supplementary Table 5. Structural alignment results for all available crystal structures corresponding to the sequence of FP-1: DRGWGNGCGLFGKGG. The results are presented as RMSD (nm) / Q_H. This alignment provided an error threshold when comparing alignments from the MD simulation with the crystal structure. That corresponded to the maximum standard deviation of aligned crystal structures (underlined in the table) which resulted in RMSD±0.05 nm and Q_H±0.07.

**PDB entry**	**1OAN**	**1OK8**	**1TG8**	**3C5X**	**3UAJ**
**1OAN**	-	0.04/0.96	0.02/0.99	0.03/0.97	0.04/0.96
**1OK8**	0.04/0.96	-	0.03/0.96	0.03/0.97	<u>0.05/0.93</u>
**1TG8**	0.02/0.99	0.03/0.96	-	0.03/0.96	0.04/0.96
**3C5X**	0.03/0.97	0.03/0.97	0.03/0.96	-	0.04/0.94
**3UAJ**	0.04/0.96	<u>0.05/0.93</u>	0.04/0.96	0.04/0.94	-

Supplementary Table 6. Structural alignment results for all available crystal structures corresponding to the sequence of FP-2: DRGWGNGCGLFGKGS. The results are presented as RMSD (nm) / Q_H. This alignment provided an error threshold when comparing alignments from the MD simulation with the crystal structure. That corresponded to the maximum standard deviation of the aligned crystal structures (underlined in the table) which resulted in RMSD±0.08 nm and Q_H±0.10.

PDB entry	3G7T	1UZG	2HG0	2I69	3I50	4FG0
**3G7T**	-	0.07/0.90	0.08/0.90	0.07/0.90	0.06/0.90	0.08/0.90
**1UZG**	0.07/0.90	-	0.07/0.92	0.04/0.96	0.05/0.96	0.07/0.91
**2HG0**	0.08/0.90	0.07/0.92	-	0.05/0.93	0.05/0.96	0.07/0.90
**2I69**	0.07/0.90	0.04/0.96	0.05/0.93	-	0.04/0.96	0.06/0.91
**3I50**	0.06/0.90	0.05/0.96	0.05/0.96	0.04/0.96	-	0.05/0.93
**4FG0**	0.08/0.90	0.07/0.91	0.07/0.90	0.06/0.91	0.05/0.93	-

Supplementary Table 7. Structural alignment results for all available crystal structures corresponding to the sequence of FP-2: DRGWGNHCGLFGKGS. The results are presented as RMSD (nm) / Q_H. This alignment provided an error threshold when comparing alignments from the MD simulation with the crystal structure.

PDB entry	1URZ
**1SVB**	0.02/0.99

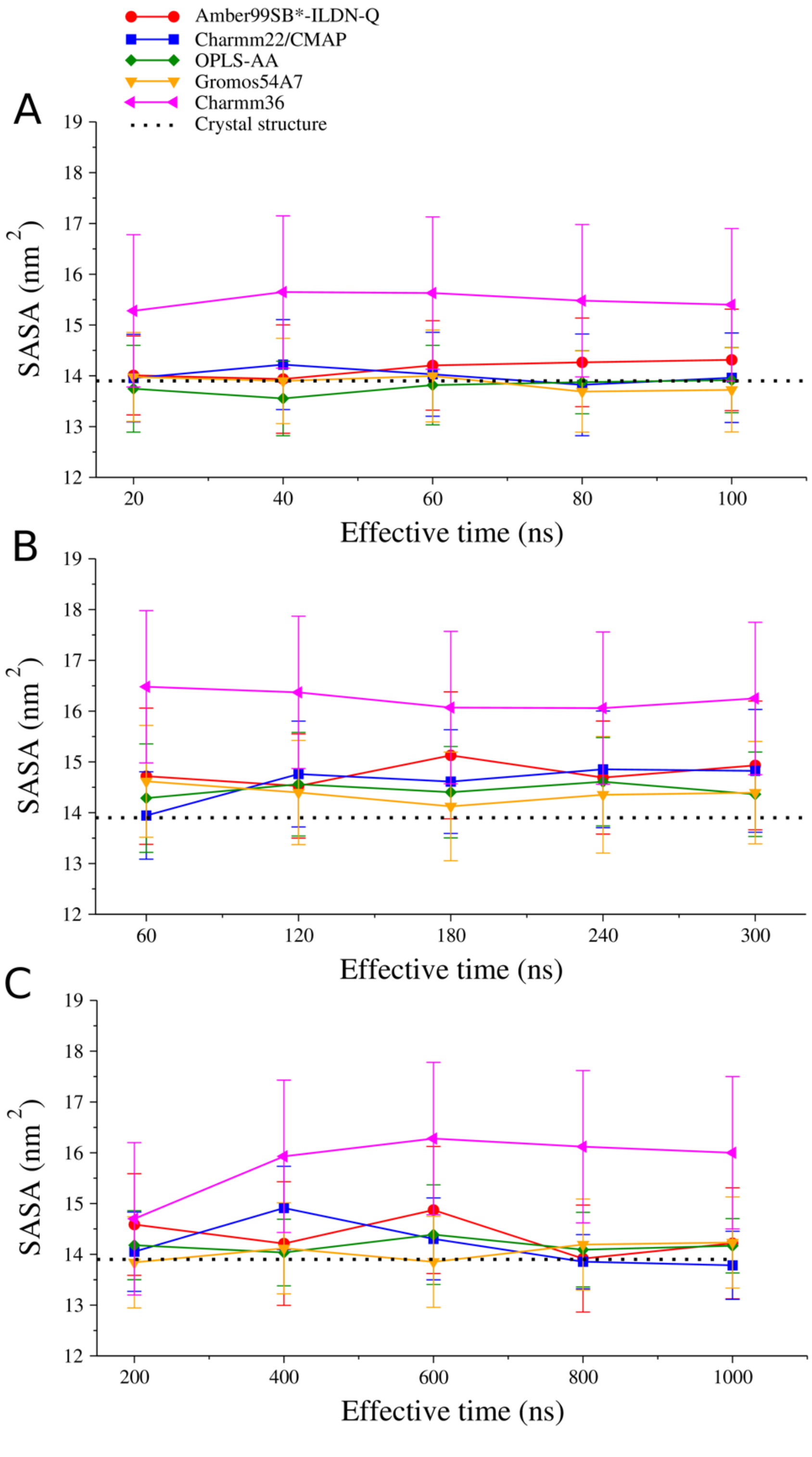

Supplementary Figure 1. Convergence analysis of the solvent accessible surface area (SASA) for the fusion peptide FP-1 simulated in explicit solvent in comparison to the crystal structure (black dotted line). Results were obtained by splitting the total effective time of given method/force field into five parts and calculating the arithmetic average for each of the effective time windows. This involved: A) Replica exchange molecular dynamics; B) Simulated annealing and C) Conventional molecular dynamics. The error bars correspond to the standard deviations.

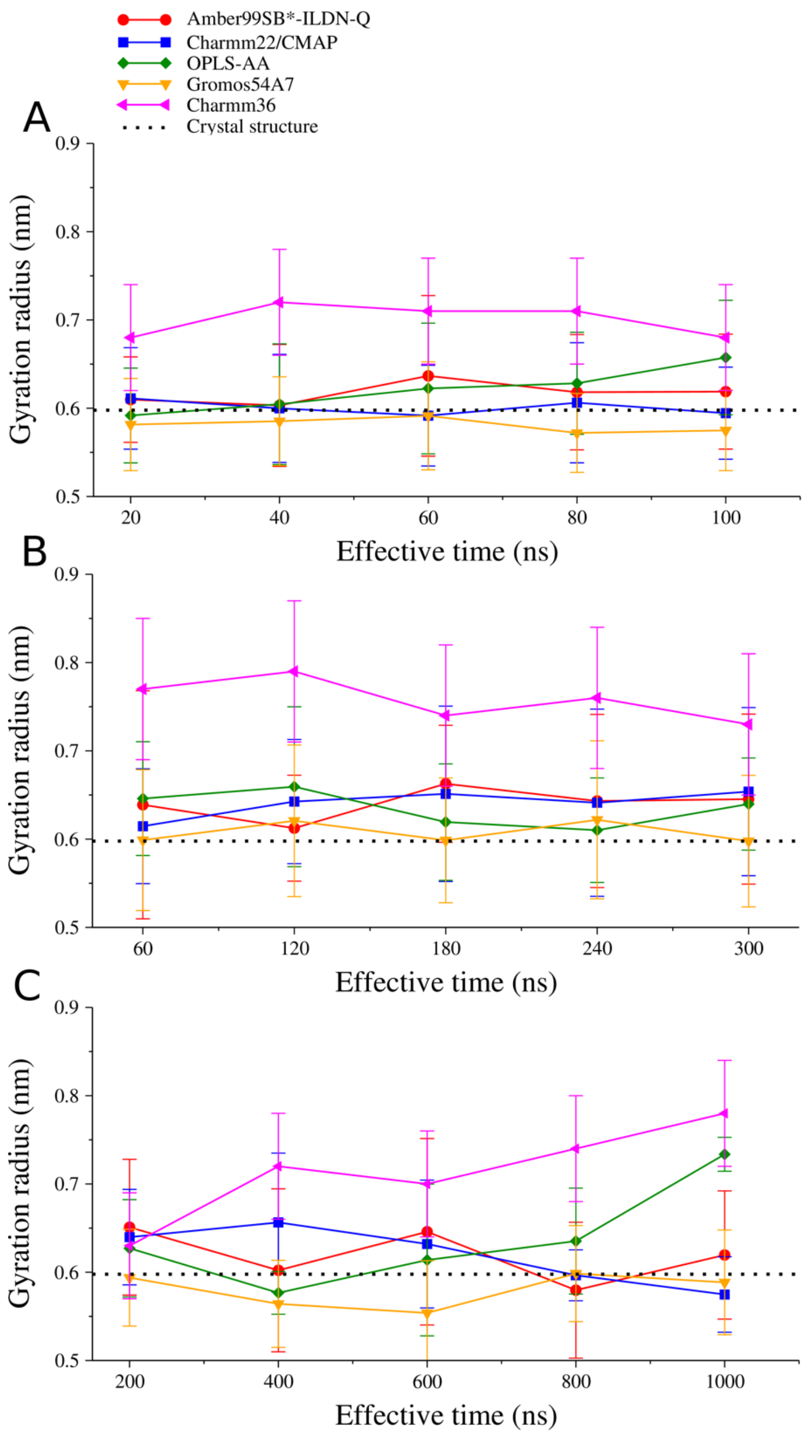

Supplementary Figure 2. Convergence analysis of the gyration radius for the fusion peptide FP-1 simulated in explicit solvent in comparison to the crystal structure (black dotted line). Results were obtained by splitting the total effective time of given method/force field into five parts and calculating the arithmetic average for each of the effective time windows. This involved: A) Replica exchange molecular dynamics; B) Simulated annealing and C) Conventional molecular dynamics. The error bars correspond to the standard deviations.

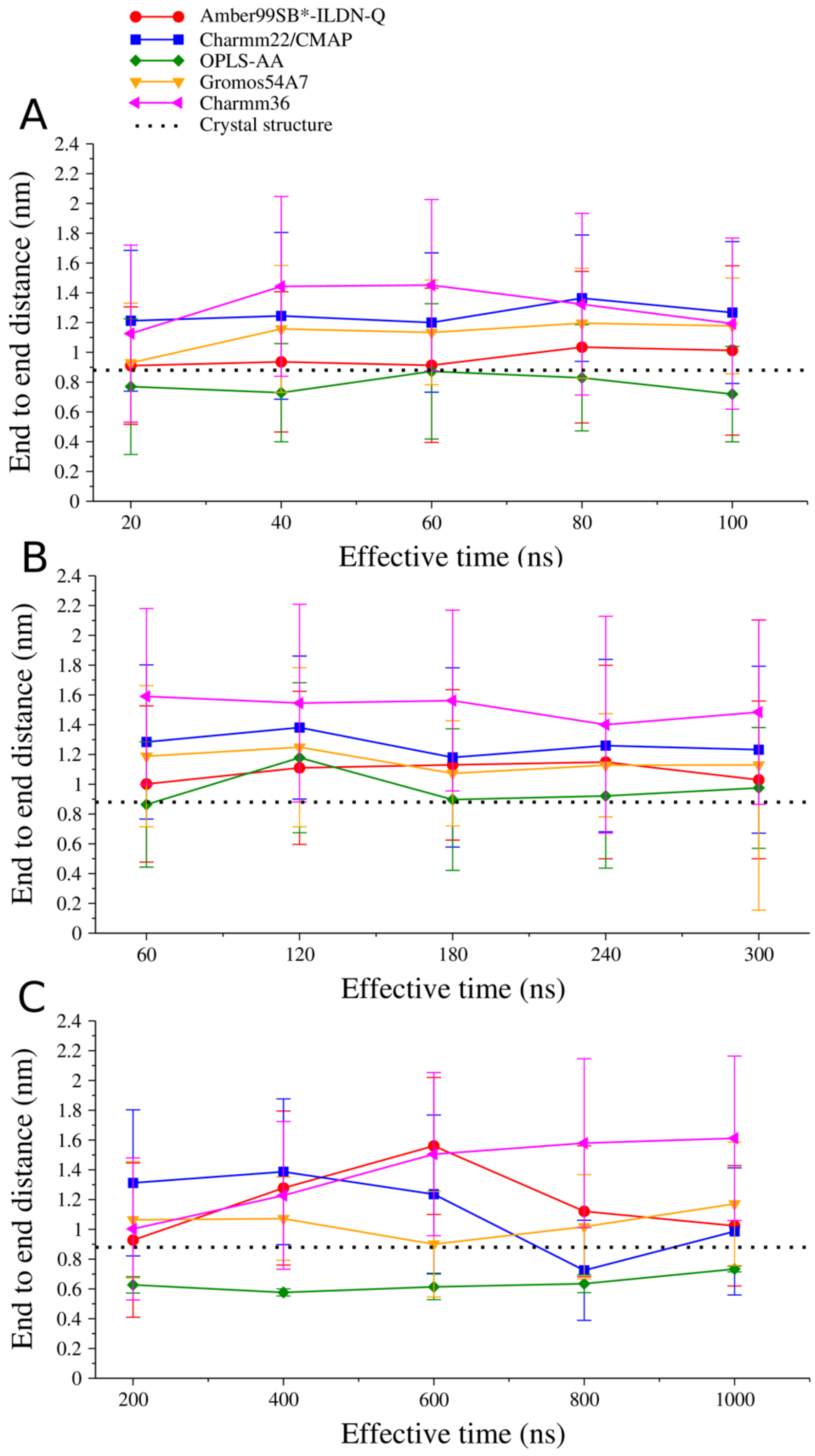

Supplementary Figure 3. Convergence analysis of the end to end distance for the fusion peptide FP-1 simulated in explicit solvent in comparison to the crystal structure (black dotted line). Results were obtained by splitting the total effective time of given method/force field into five parts and calculating the arithmetic average for each of the effective time windows. This involved: A) Replica exchange molecular dynamics; B) Simulated annealing and C) Conventional molecular dynamics. The error bars correspond to the standard deviations.

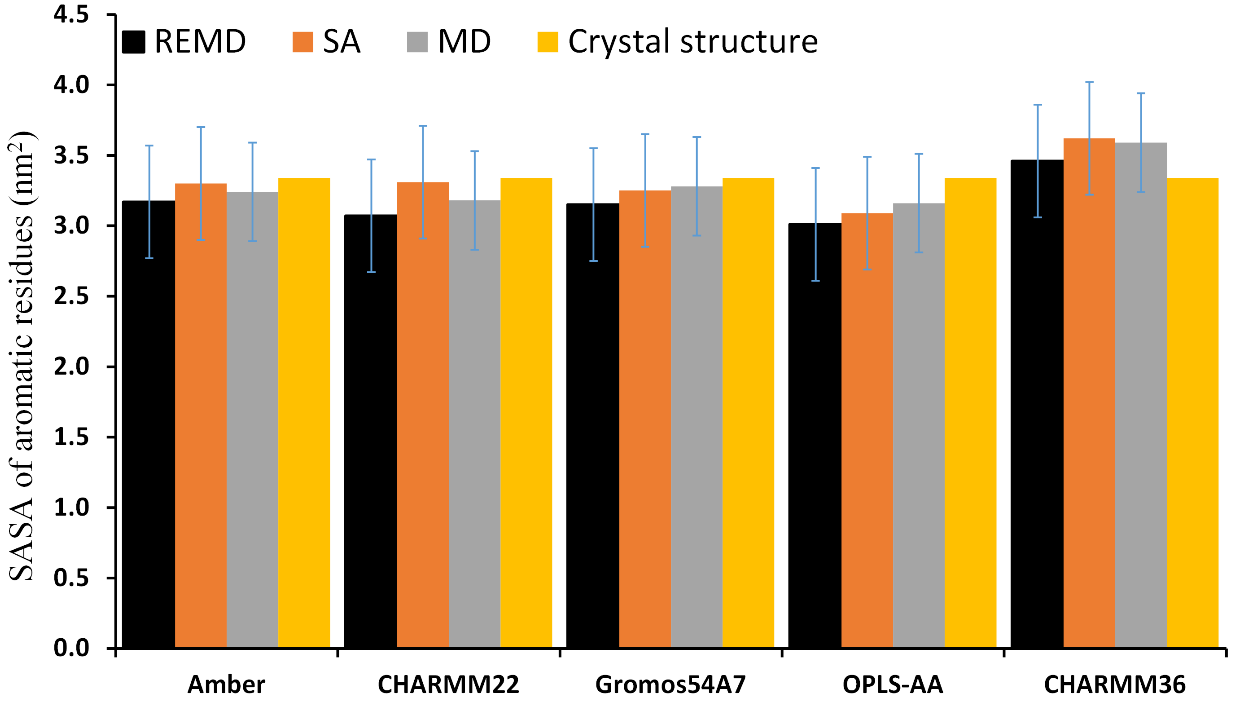

Supplementary Figure 4. Solvent accessible surface area (SASA) of the aromatic residues, namely W101 and F108 obtained for the crystal structure (yellow), REMD (blue), SA (orange), and conventional MD (grey). Data is shown for four force fields: Amber99SB*-ILDN-Q, Charmm22/CMAP, Gromos54A7 and OPLS-AA. Error bars correspond to standard deviations.

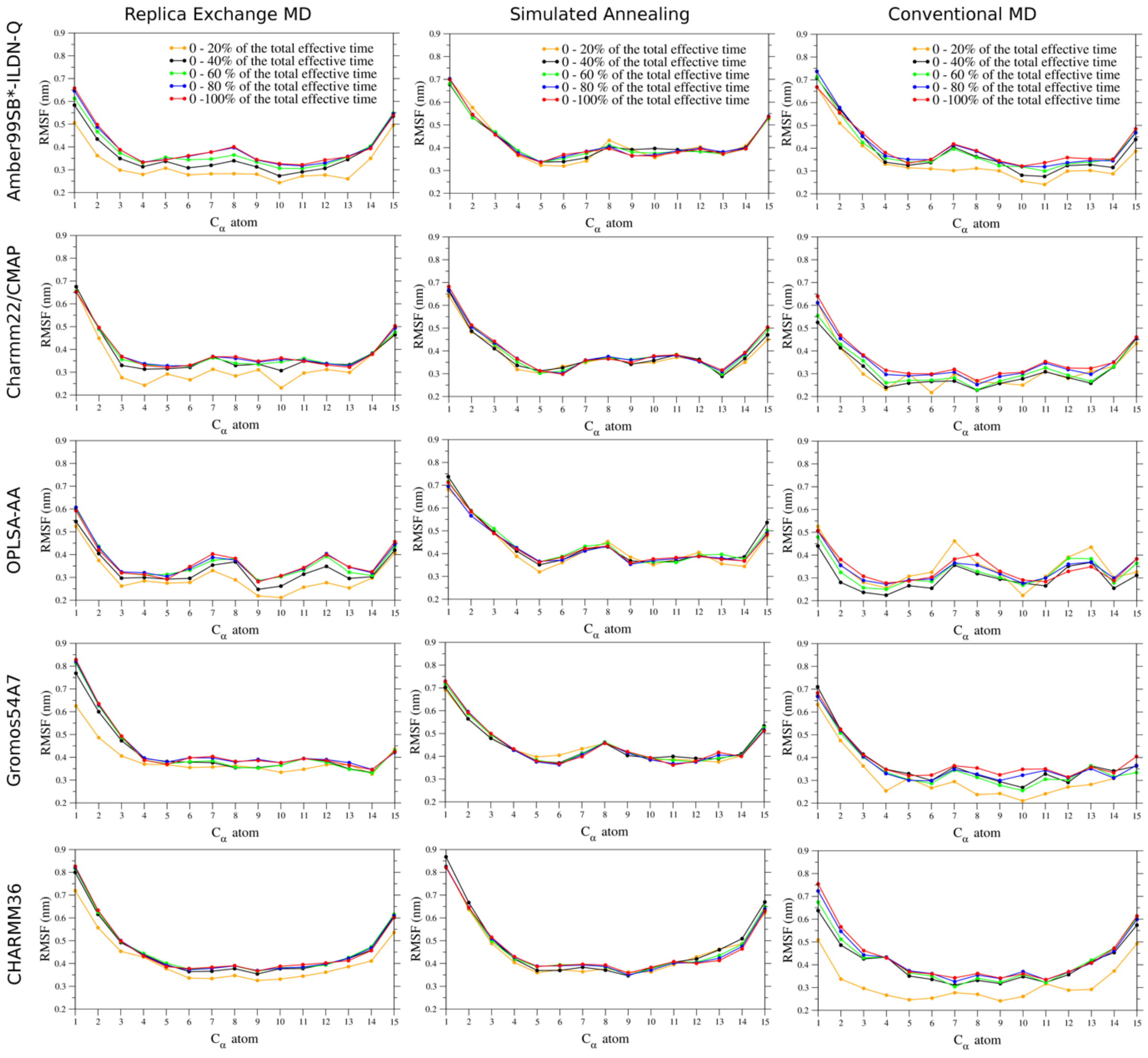

Supplementary Figure 5. Root mean square fluctuation (RMSF) of Cα atoms averaged over 0-20%, 0-40%, 0-60% and 0-100% of the total simulation time for each employed method and force field.

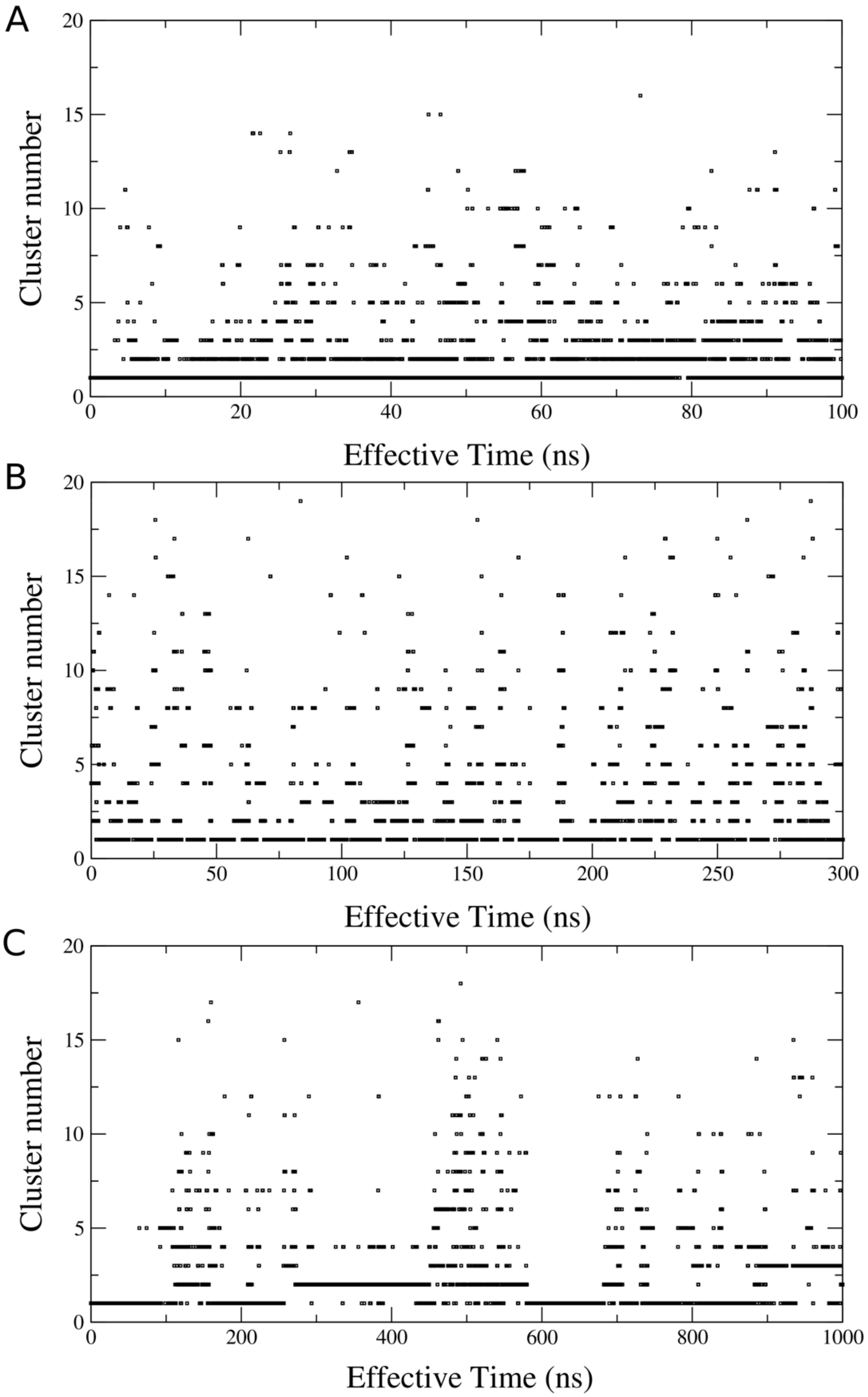

Supplementary Figure 6. Clustering analysis results for FP-1 simulations. These were performed using Amber99SB*-ILDN-Q and: A) replica exchange molecular dynamics (REMD), B) simulated annealing (SA) and C) conventional molecular dynamics (MD), presented as the cluster number versus the simulation time.

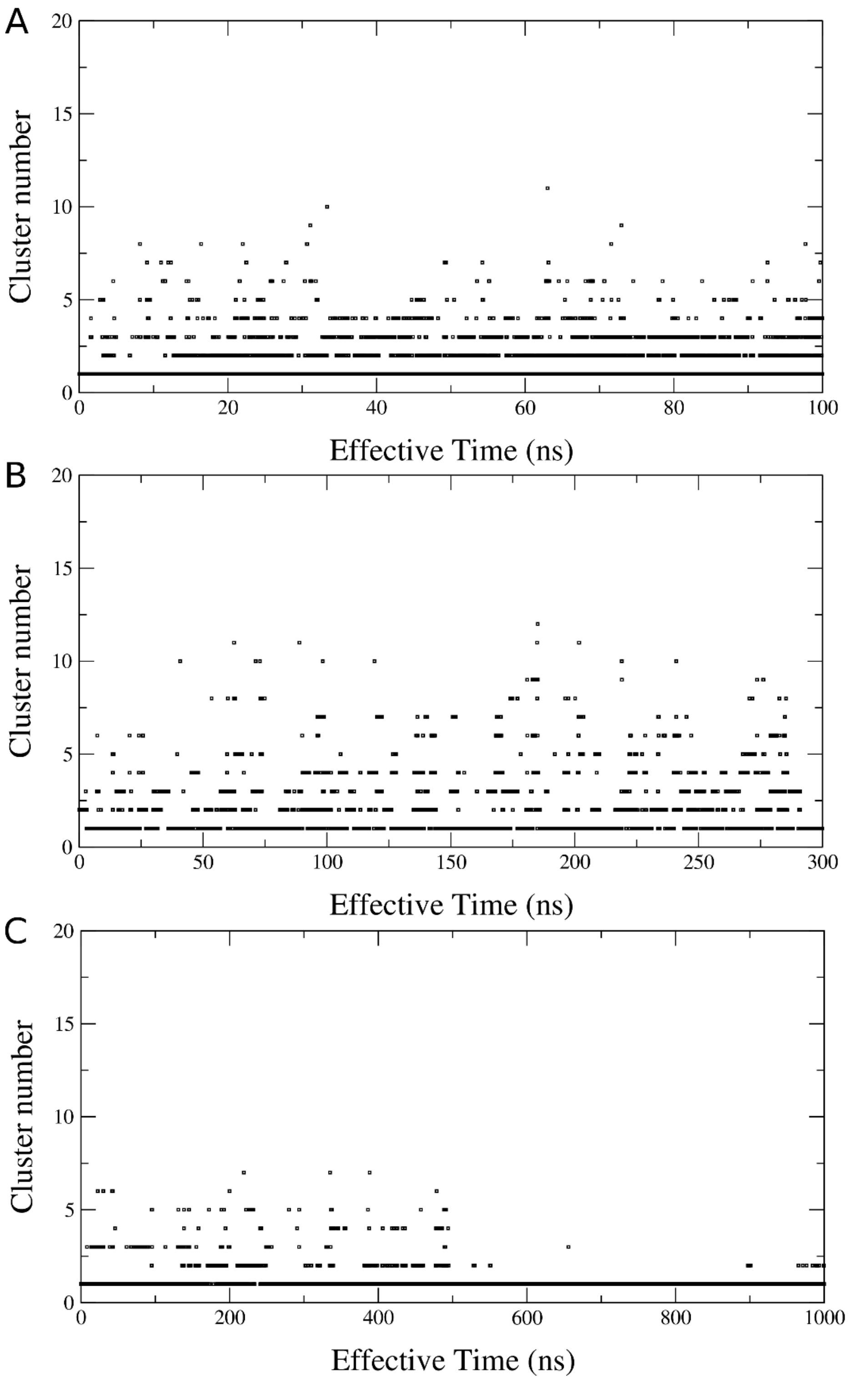

Supplementary Figure 7. Clustering analysis results for FP-1 simulations. These were performed using Charmm22/CMAP and: A) replica exchange molecular dynamics (REMD), B) simulated annealing (SA) and C) conventional molecular dynamics (MD), presented as the cluster number versus the simulation time.

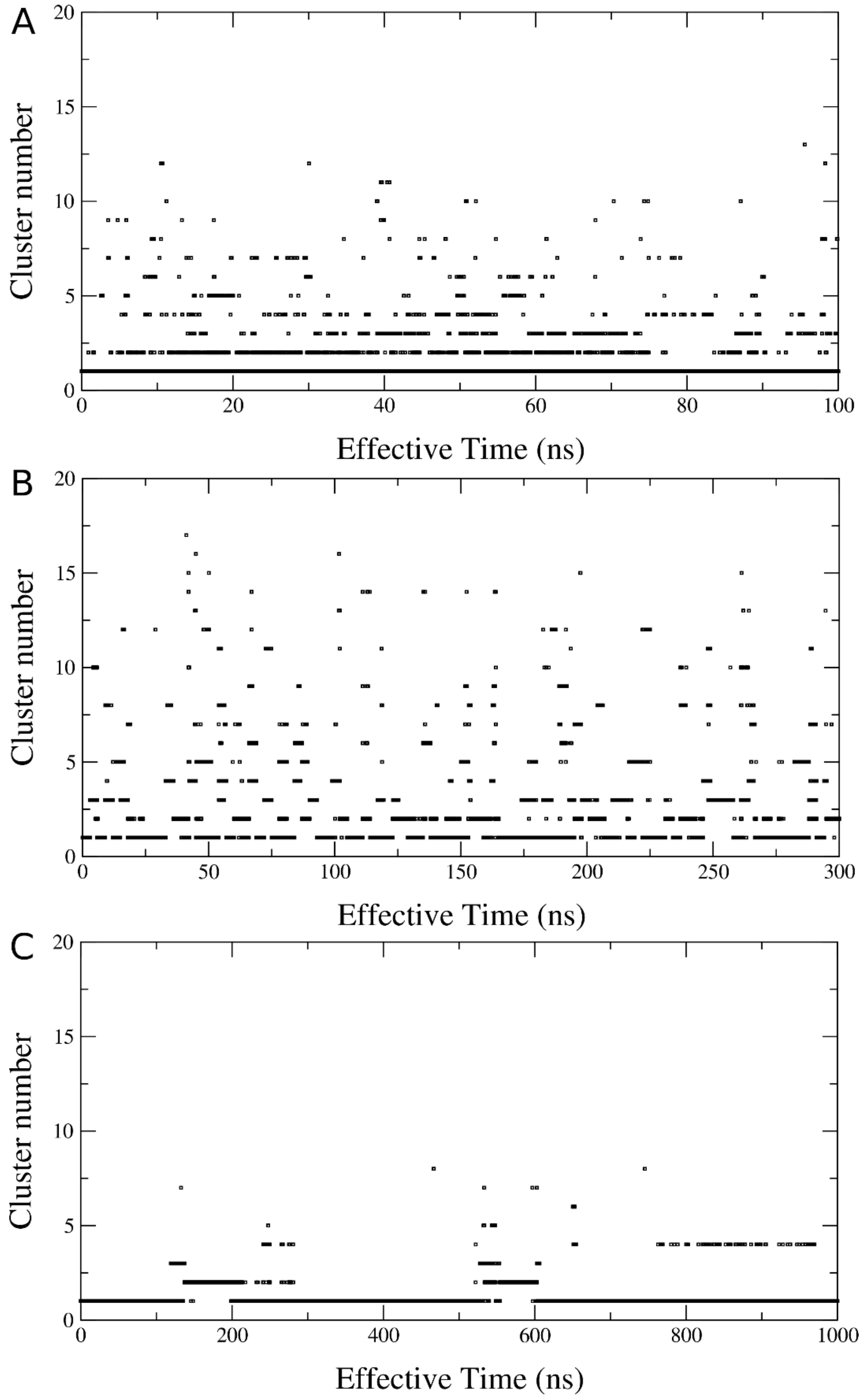

Supplementary Figure 8. Clustering analysis results for FP-1 simulations. These were performed using OPLS-AA and: A) replica exchange molecular dynamics (REMD), B) simulated annealing (SA) and C) conventional molecular dynamics (MD), presented as the cluster number versus the simulation time.

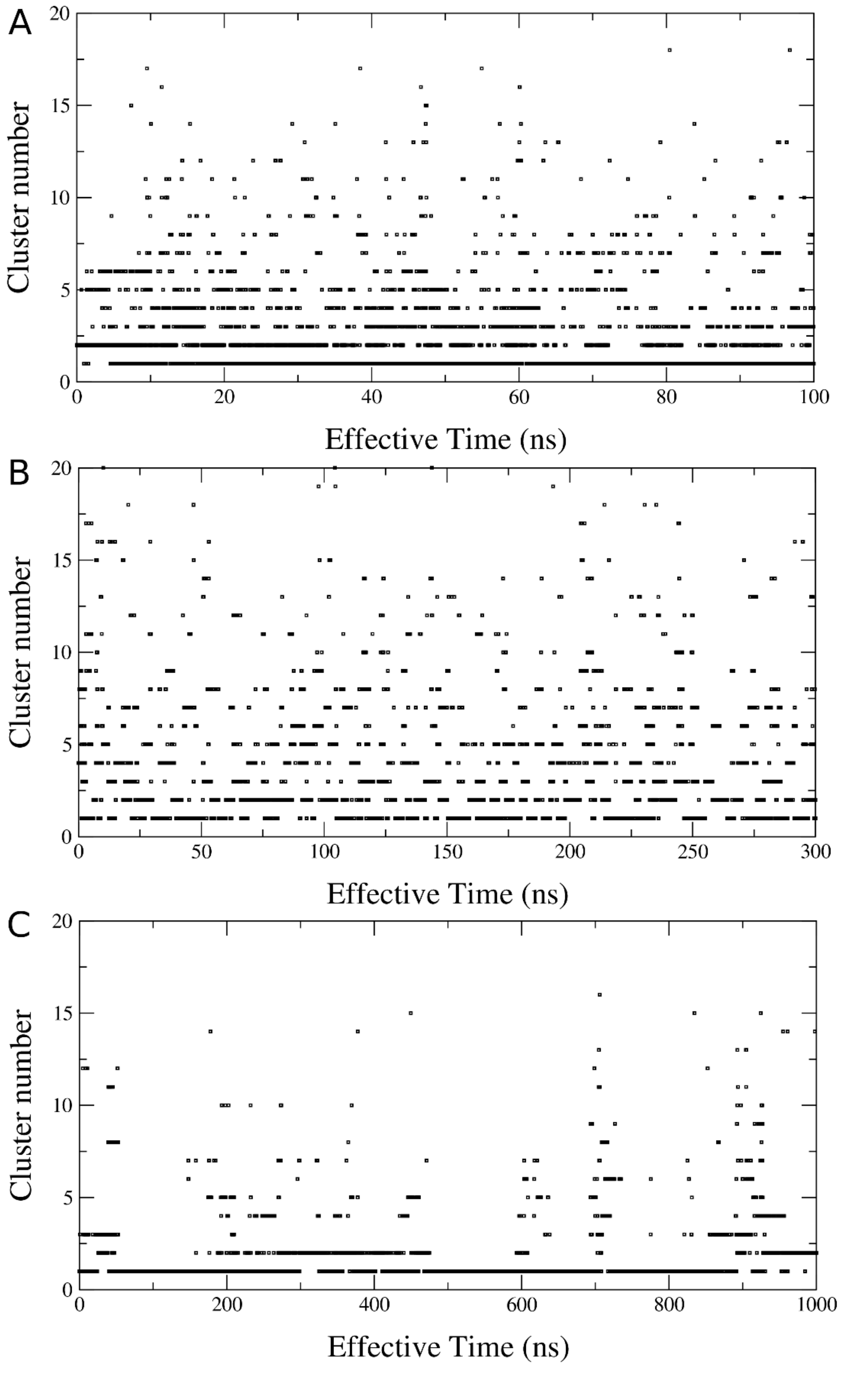

Supplementary Figure 9. Clustering analysis results for FP-1 simulations. These were performed using Gromos54A7 and: A) replica exchange molecular dynamics (REMD), B) simulated annealing (SA) and C) conventional molecular dynamics (MD), presented as the cluster number versus the simulation time.

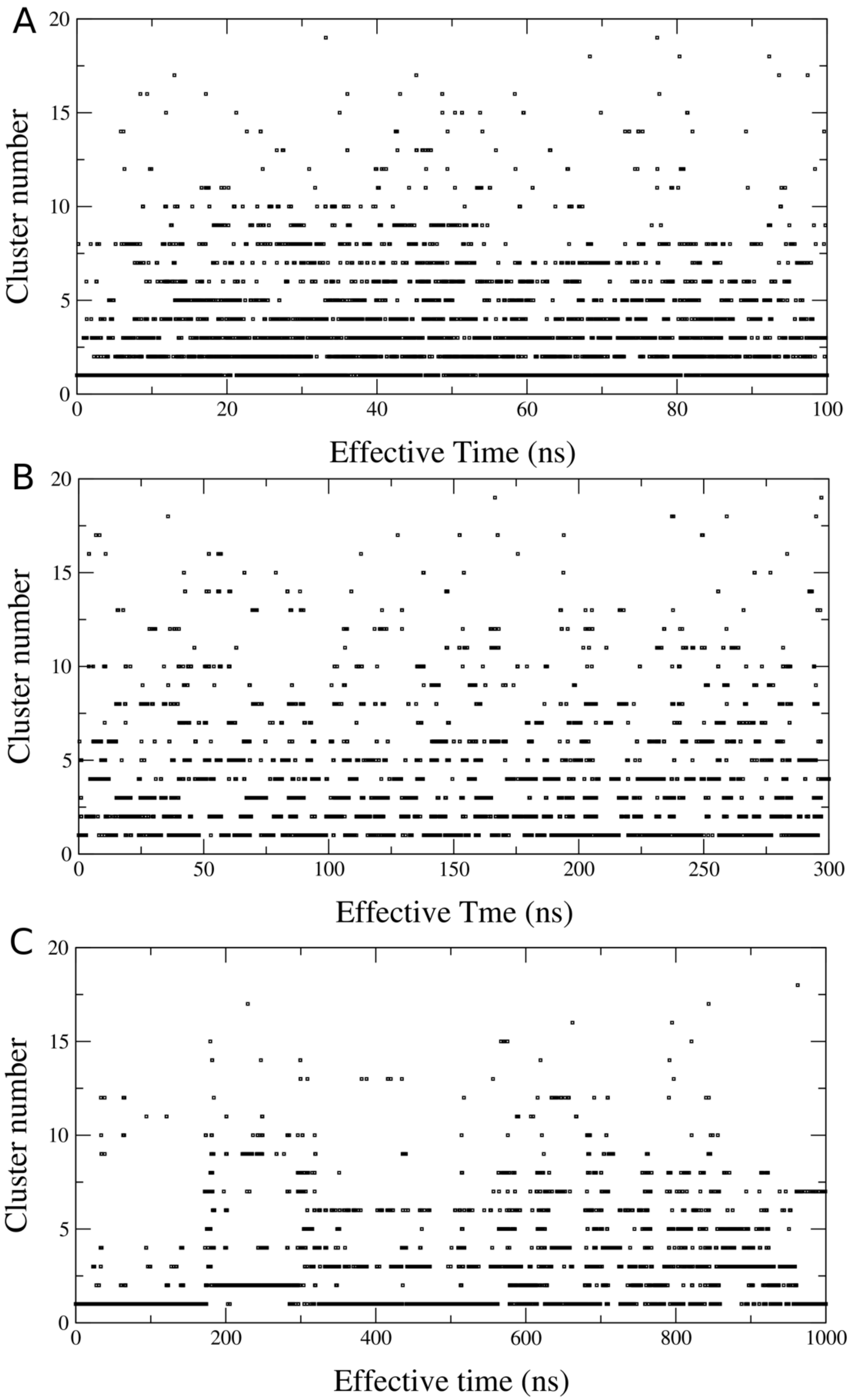

Supplementary Figure 10. Clustering analysis results for FP-1 simulations. These were performed using CHARMM36 and: A) replica exchange molecular dynamics (REMD), B) simulated annealing (SA) and C) conventional molecular dynamics (MD), presented as the cluster number versus the simulation time.

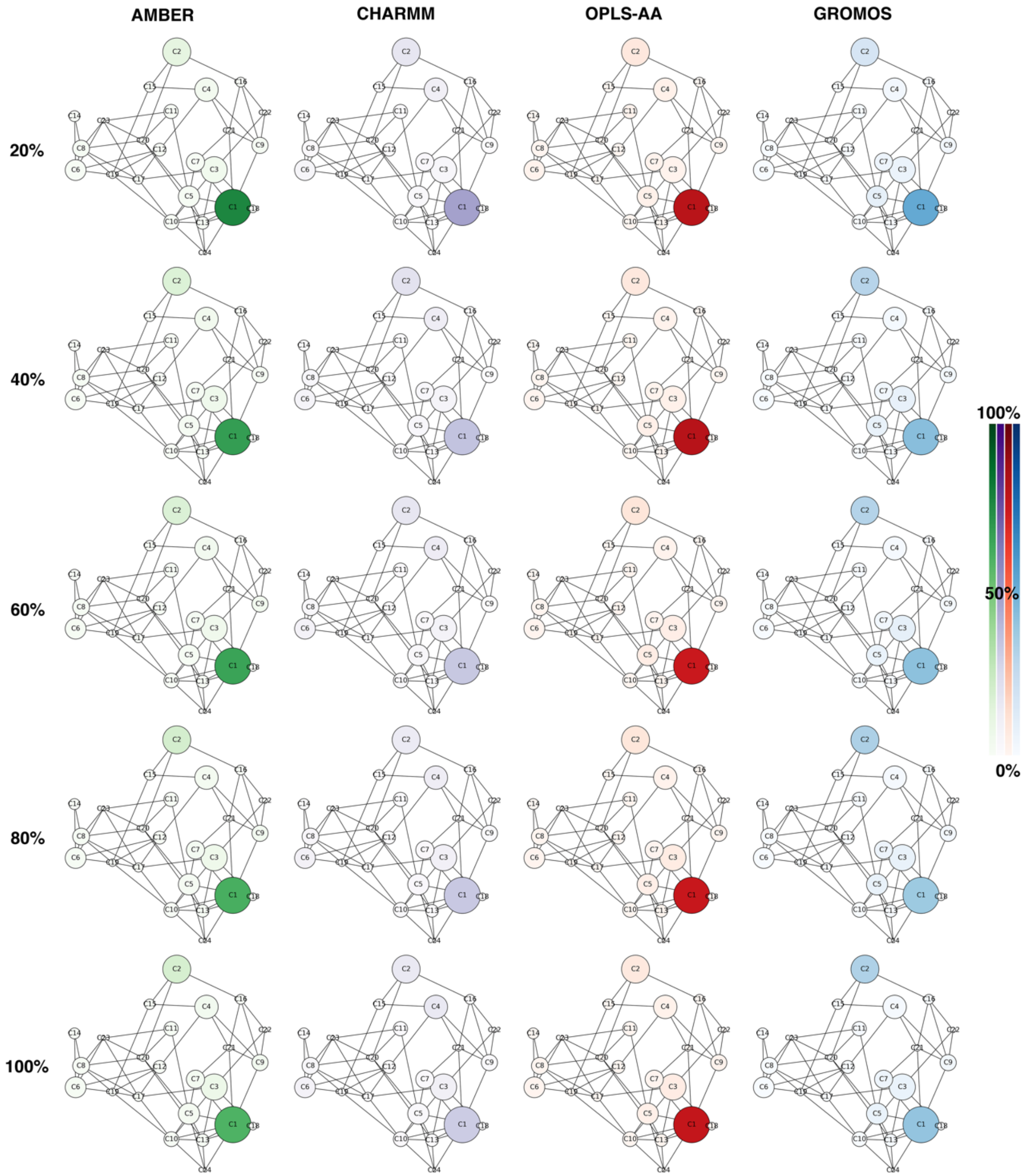

Supplementary Figure 11. Time evolution of cluster populations using replica exchange molecular dynamics (REMD). Starting in cluster 1, REMD simulations sample the rest of the conformational network over time. Sampling is particularly effective for the CHARMM36 and Gromos57A4 force fields, while the Amber ff99SB*-ILDN-Q and OPLS-AA force fields remain more focused on cluster 1. Colours indicate relative populations of individual clusters using a specific method and force field.

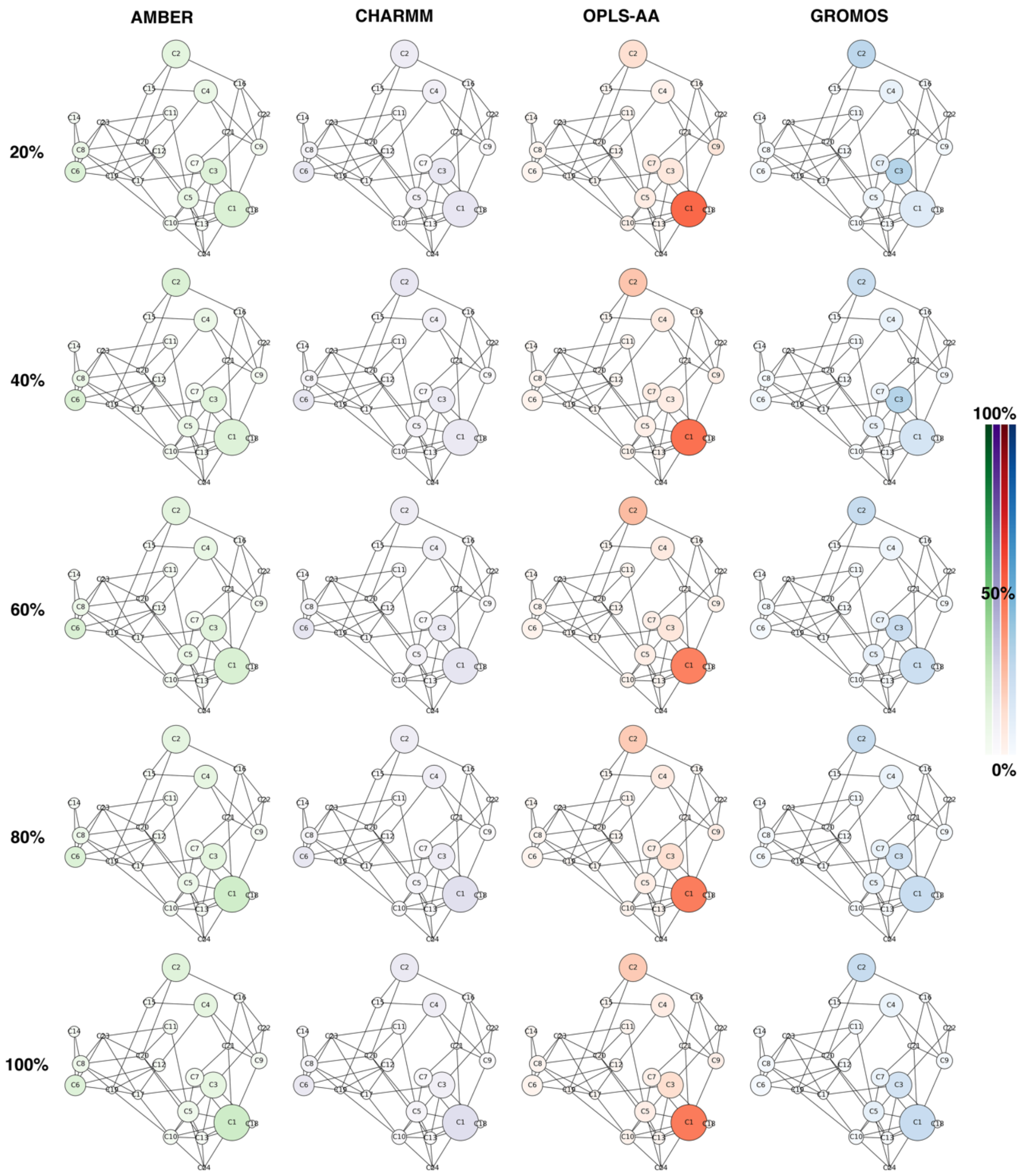

Supplementary Figure 12. Time evolution of cluster populations using simulated annealing (SA) which yields quick sampling of the available conformational space. At 20% of the total simulation time, significant conformational diversity is apparent for all investigated force fields. Colours indicate relative populations of individual clusters using a specific method and force field.

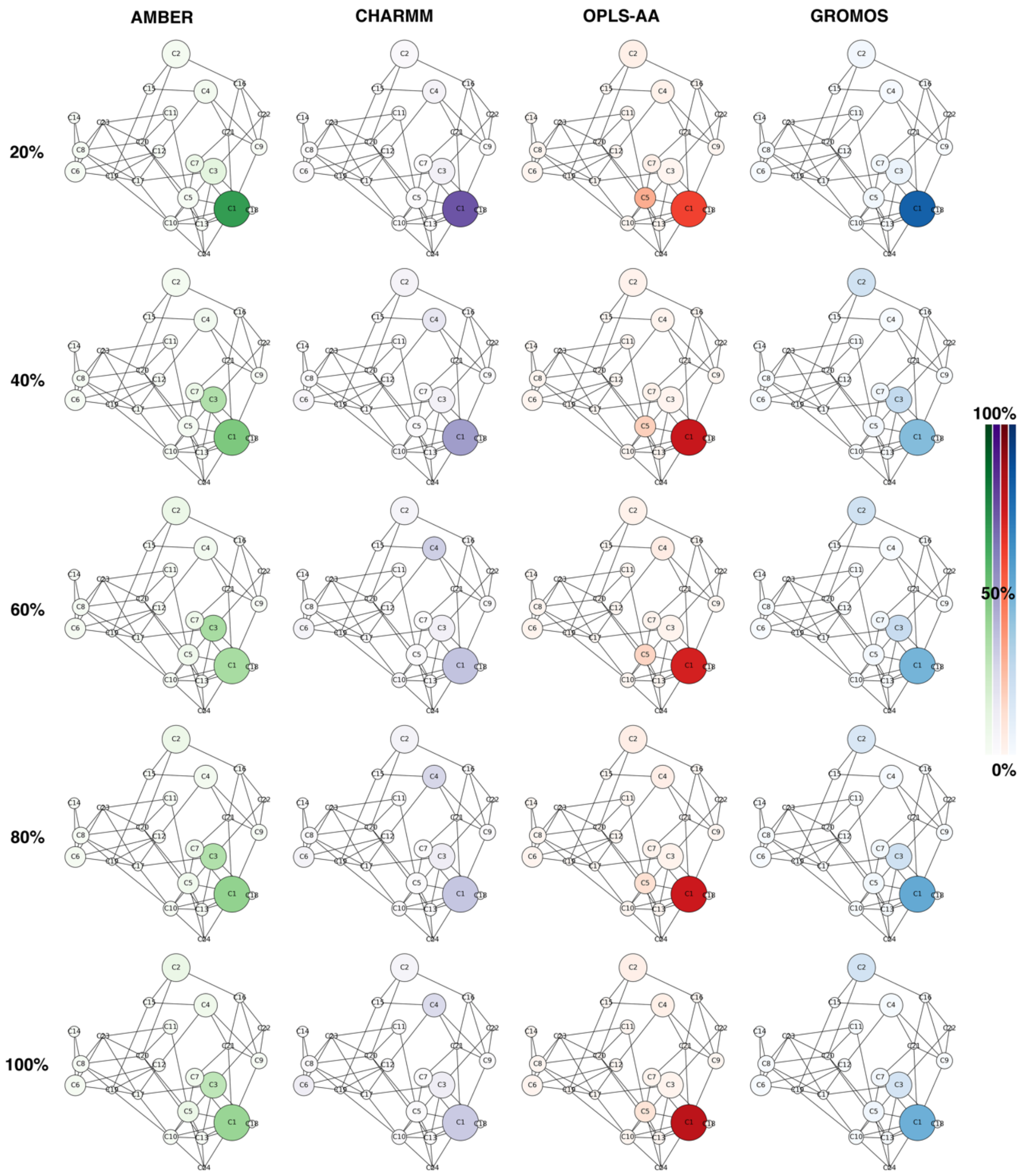

Supplementary Figure 13. Time evolution of cluster populations using conventional molecular dynamics (MD). Conventional MD simulation shows a pattern of slower conformational sampling than either replica exchange MD (REMD) or simulated annealing (SA) simulations. As the initial concentration within cluster 1 is reduced, closely related clusters are populated first. Interestingly, the focus on cluster 1 increases for the OPLS-AA force field. Colours indicate relative populations of individual clusters using a specific method and force field.

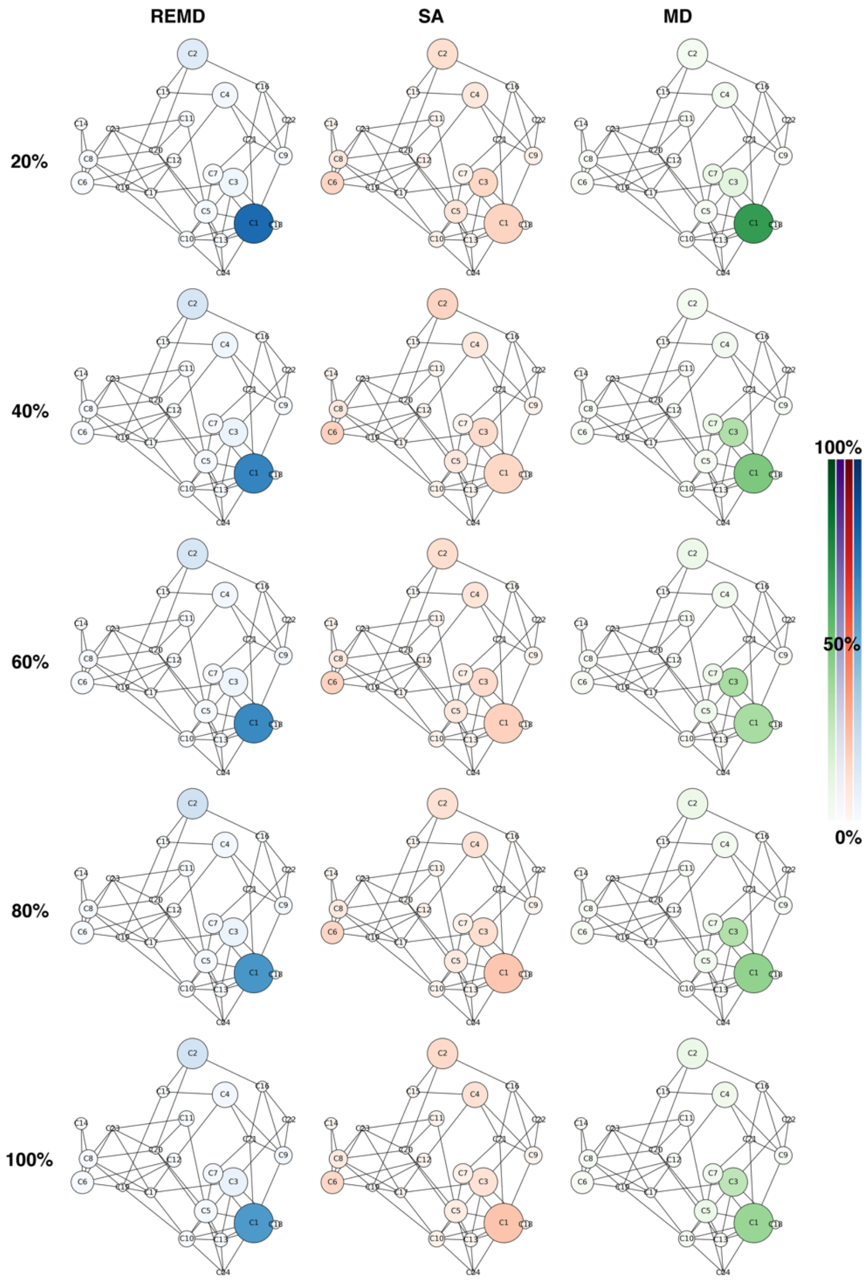

Supplementary Figure 14. Time evolution of cluster populations using the Amber99SB*-ILDN-Q force field. Simulated annealing offers a diverse conformational sampling. Interestingly, REMD yields higher populations for cluster 1, whereas conventional MD shows concentration in structurally related clusters around cluster 1. Colours indicate relative populations of individual clusters using a specific method and force field.

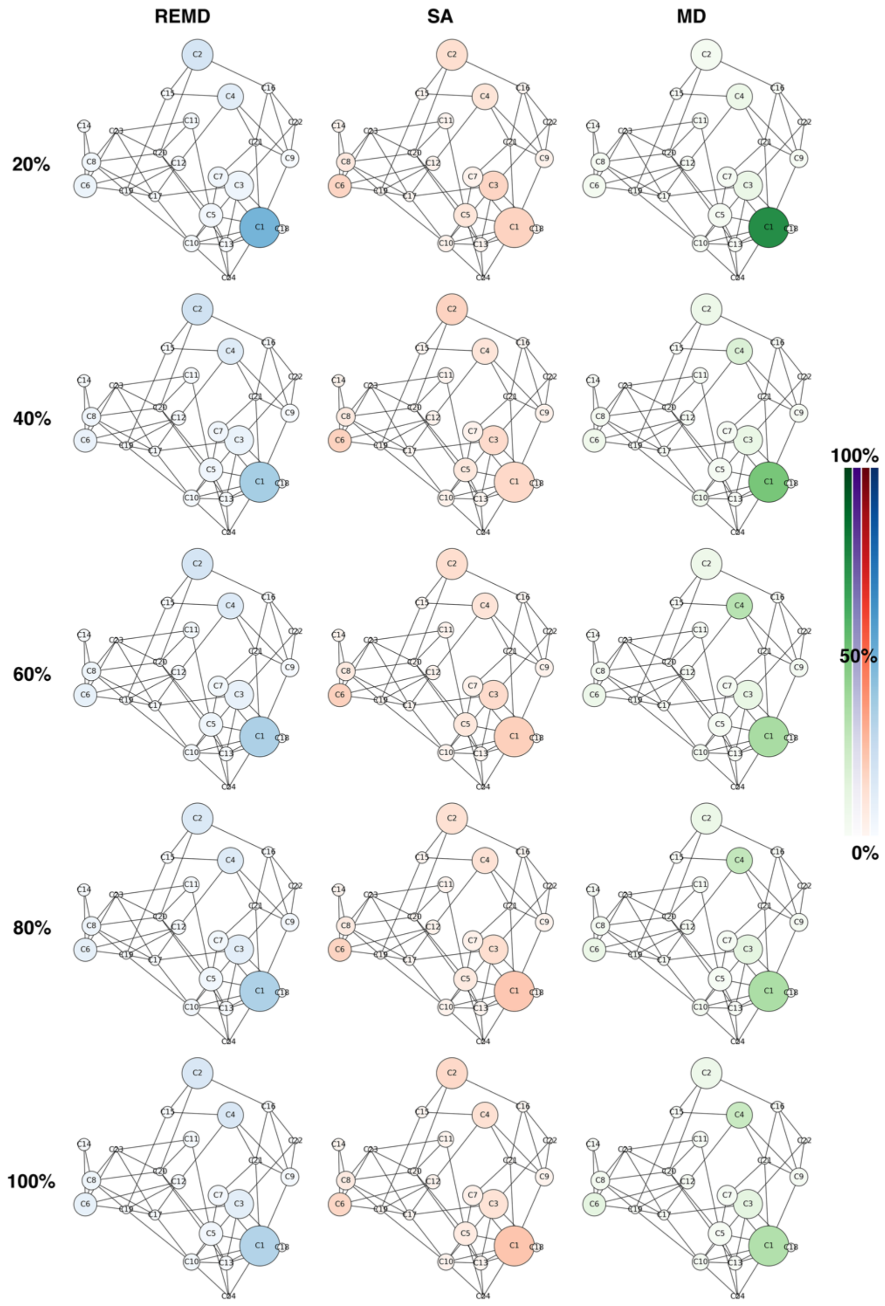

Supplementary Figure 15. Time evolution of cluster populations using the CHARMM36 force field. The CHARMM36 force field offers the broadest conformational ensemble irrespective of sampling method used. Ensembles between simulated annealing and replica exchange molecular dynamics very similar, whereas conventional molecular dynamics exhibits some residual concentration in closely related clusters. Colours indicate relative populations of individual clusters using a specific method and force field.

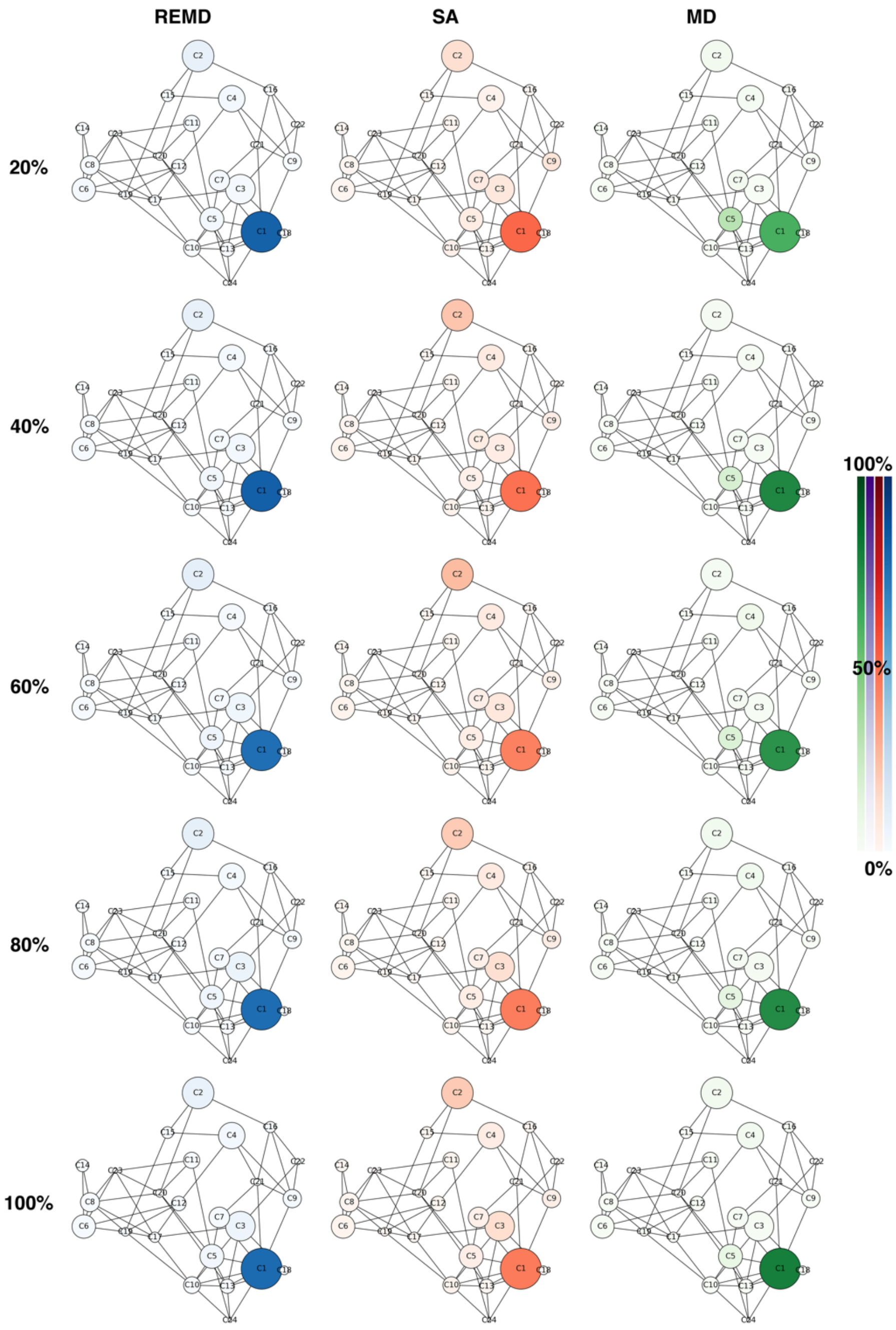

Supplementary Figure 16. Time evolution of cluster populations using the OPLS-AA force field. The OPLS-AA force field is focused on the cluster 1 conformation. All sampling methods show a strong concentration of observed states in this cluster. Simulated annealing is most efficient at exploring different clusters in this force field. Colours indicate relative populations of individual clusters using a specific method and force field.

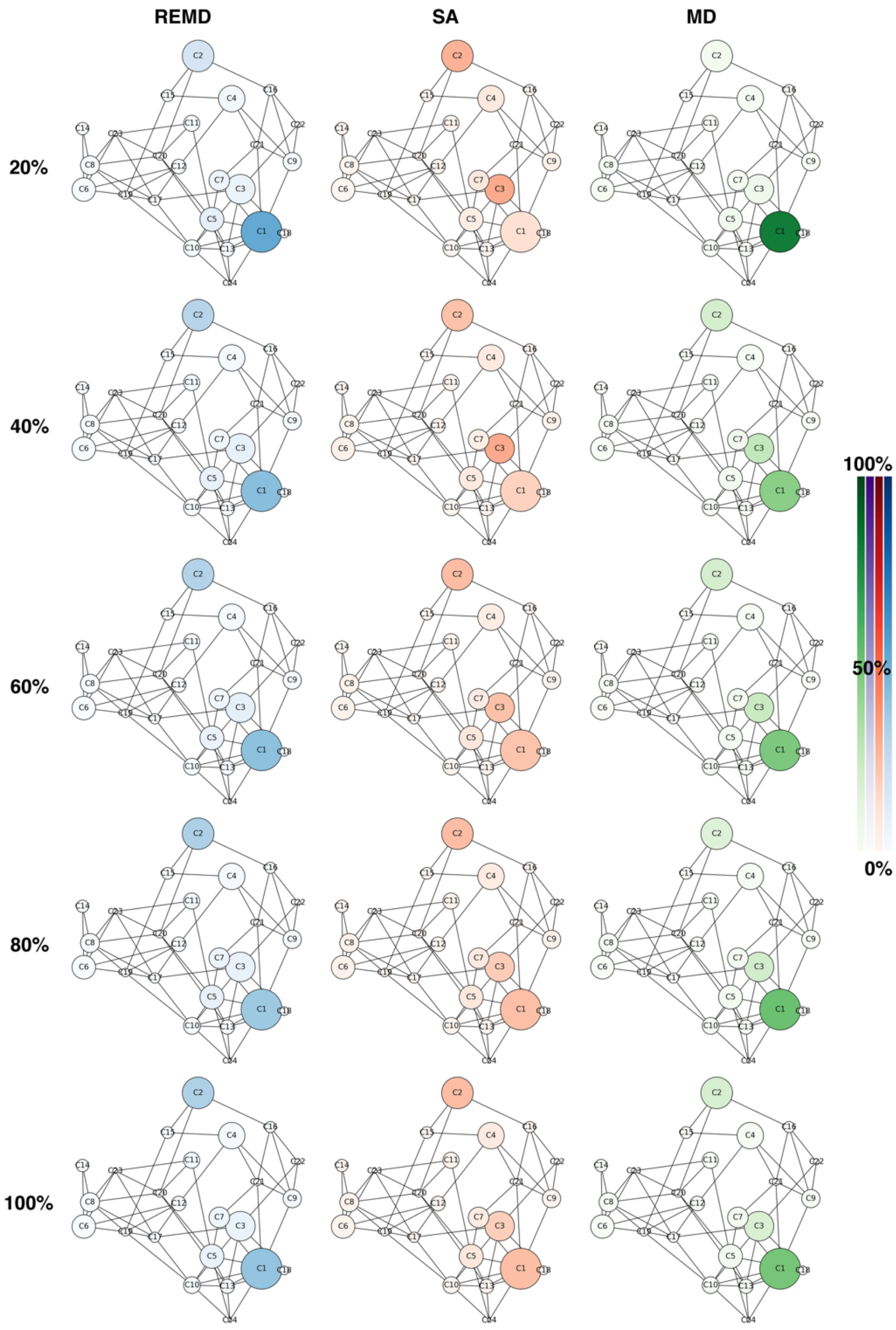

Supplementary Figure 17. Time evolution of cluster populations using the Gromos57A4 force field. All sampling methods within the Gromos57A4 force field yield similar cluster populations. MD is slightly less efficient than SA to converge to population equilibrium. Colours indicate relative populations of individual clusters using a specific method and force field.

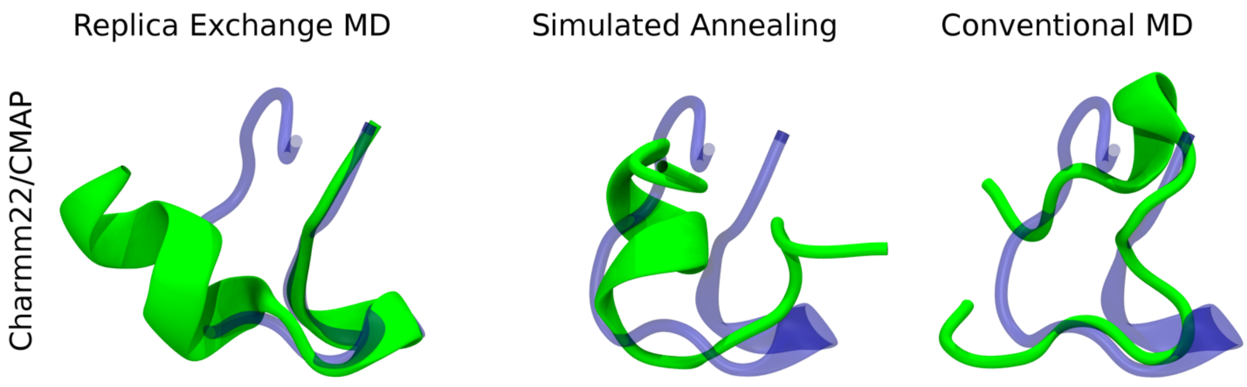

Supplementary Figure 18. Comparison of structures generated by simulation with CHARMM22/CMAP forcefield and experiment. Representative snapshots are shown for the first cluster of FP-1 explored by replica exchange molecular dynamics, simulated annealing and conventional molecular dynamics (MD) using CHARMM22/CMAP. The peptide is shown in cartoon representation for the crystal structure (PDB: 1OAN, blue) and CHARMM22/CMAP (green).

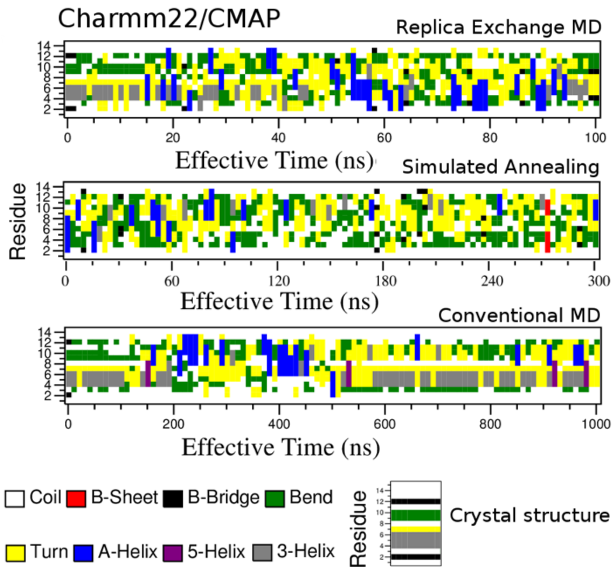

Supplementary Figure 19. Secondary structure analysis of FP-1 by the use of CHARMM22/CMAP. Propensity for per-residue secondary structural features as a function of simulation time, shown for all three sampling methods. For reference, the structural features of the crystal structure are shown at the bottom.

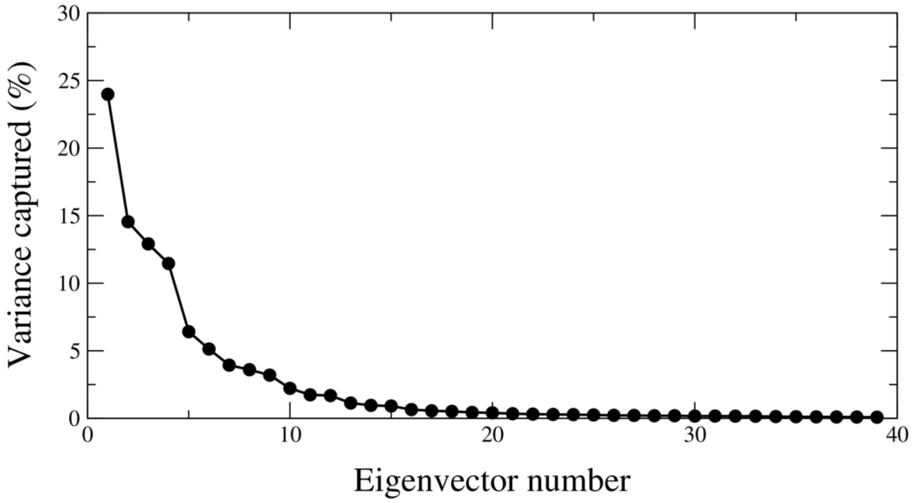

Supplementary Figure 20. Percentage fraction of total variance captured by each principal component of the combined trajectory involving all methods (REMD, SA, MD) and all force fields (Amber, Charm36, OPLS-AA, Gromos).

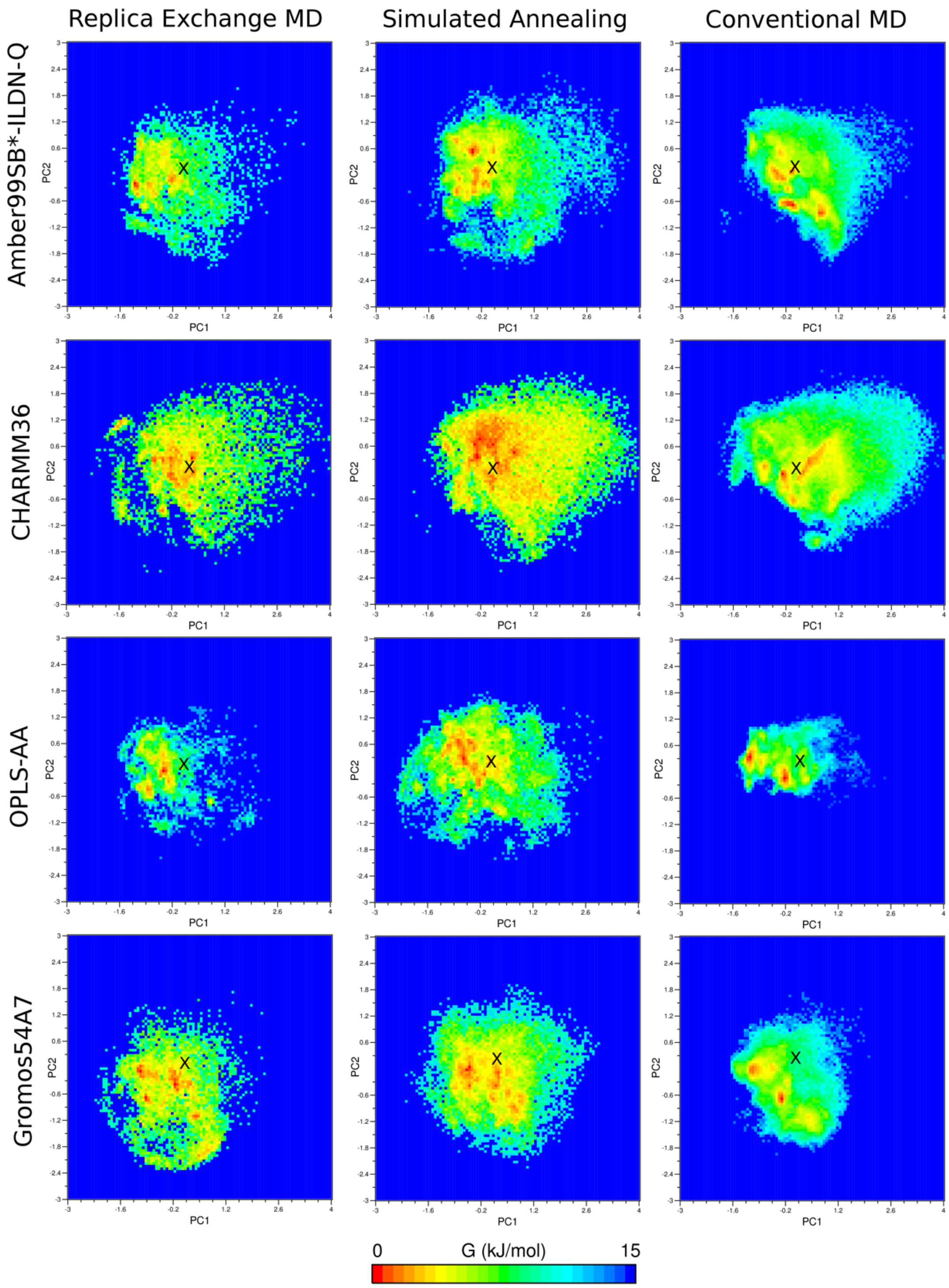

Supplementary Figure 21. Energy landscape analysis for pairs of sampling method and FF. Contour plots representing the free-energy landscapes of FP-1 as a function of the first (PC1) and second (PC2) principal components are shown, comparing combinations of sampling methods and force fields. The location of the crystal structure on each plot is denoted with a cross. The principal components were extracted from the combined trajectory of all methods and force fields (FFs) and then eigenvectors were projected using specific method/FF.

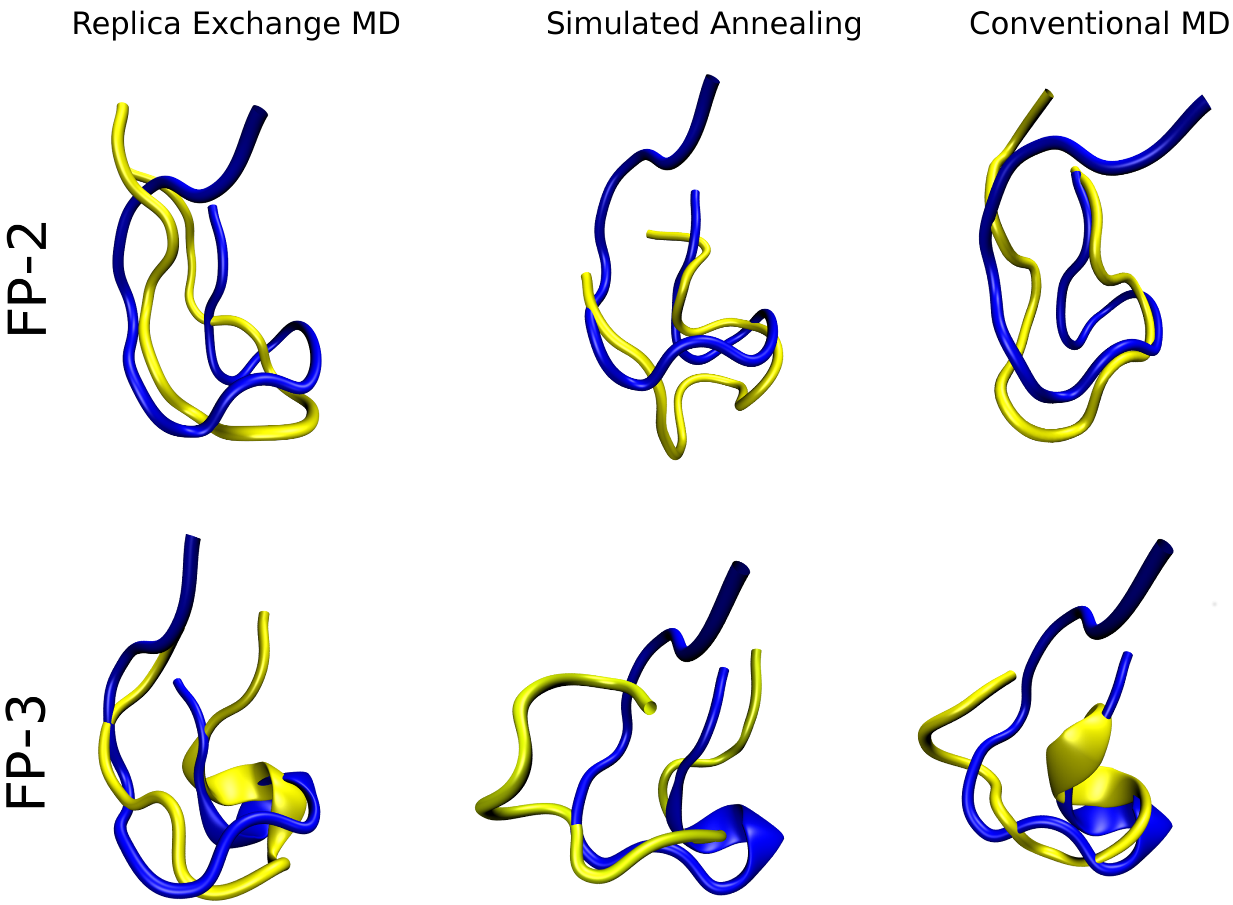

Supplementary Figure 22. Representative snapshots of the most populated conformation of the fusion peptide FP-2 and FP-3 explored by various methods: replica exchange molecular dynamics (REMD), simulated annealing (SA) and conventional molecular dynamics (MD) with the Amber99SB*-ILDN-Q force field. The protein is shown in cartoon representation, with blue indicating the respective crystal structure (FP-2, PDB:1UZG; FP-3, PDB:1URZ), and yellow indicating the most populated cluster obtained from simulation.

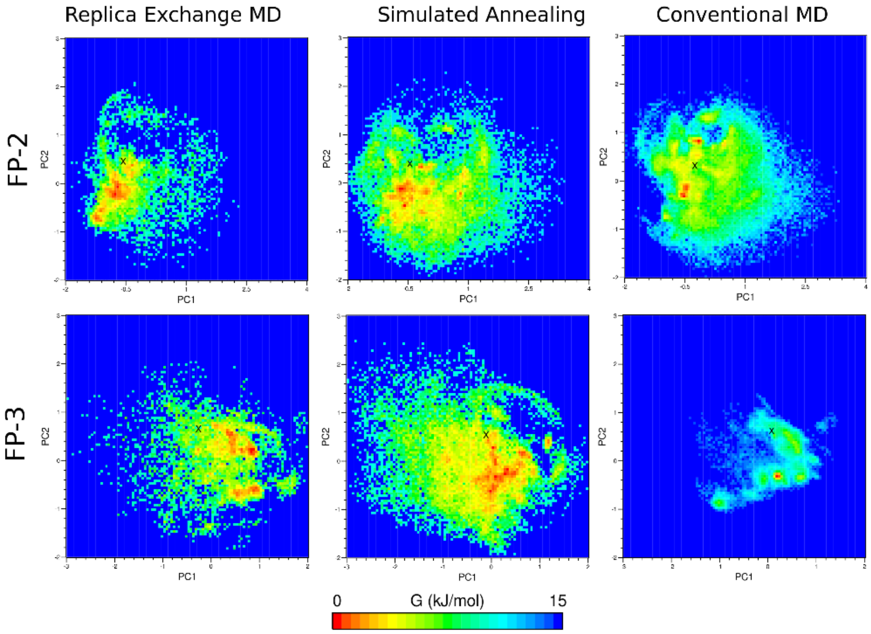

Supplementary Figure 23. Free-energy landscapes of the fusion peptides FP-2 and FP-3, assessed by principal component analysis (PCA), defined by the first and second component for each system using various methods and force fields. The location of the crystal structure on each plot is denoted with a cross. The principal components were extracted from the combined trajectory of all methods and force fields (FFs) and then eigenvectors were projected using specific method/FF.

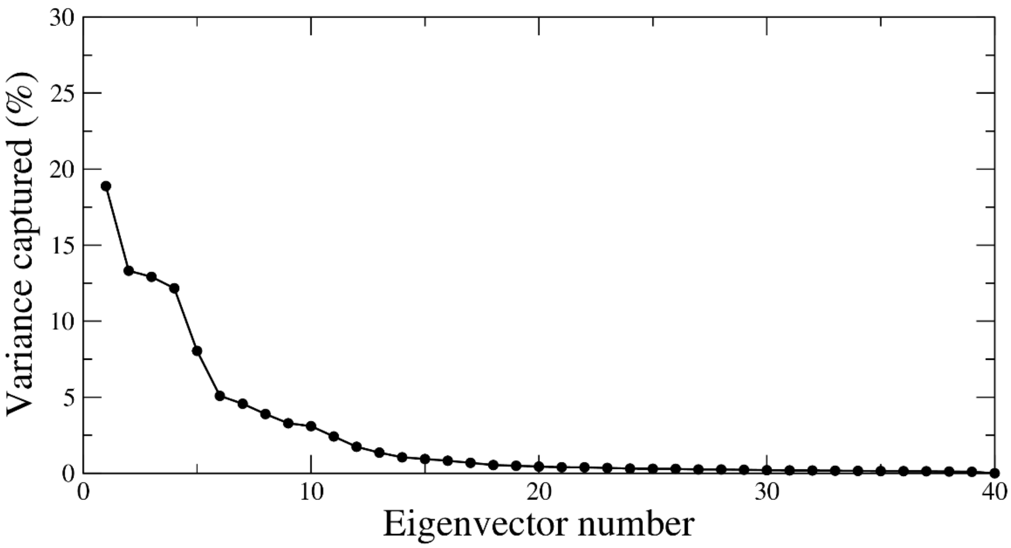

Supplementary Figure 24. Percentage fraction of the total variance captured by each principal component of the combined trajectory involving all fusion peptides: FP-1, FP-2, FP-3, FP4, using REMD/Amber99SB*-ILDN-Q.

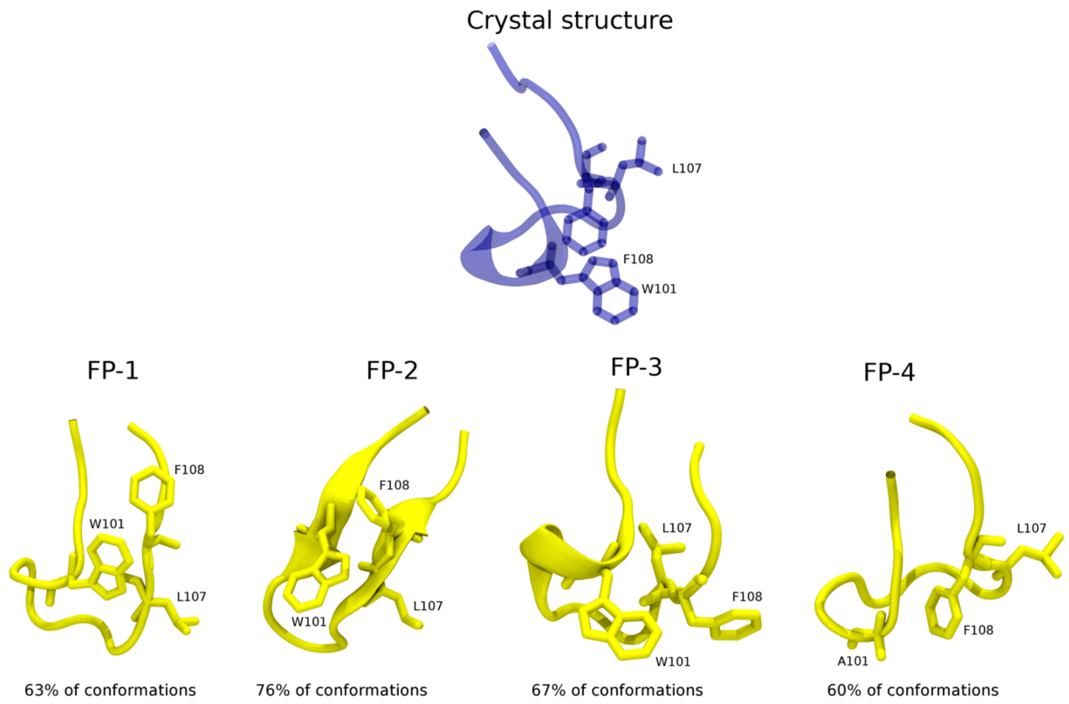

Supplementary Figure 25. The most dominant conformations as a result of clustering analysis for all fusion peptides studied by the application of replica exchange molecular dynamics and Amber99SB*-ILDN-Q. The hairpin structure is shared by all fusion peptides with three (W101 or A101, L107 and F108) hydrophobic residues packed together, but in variable orientations.

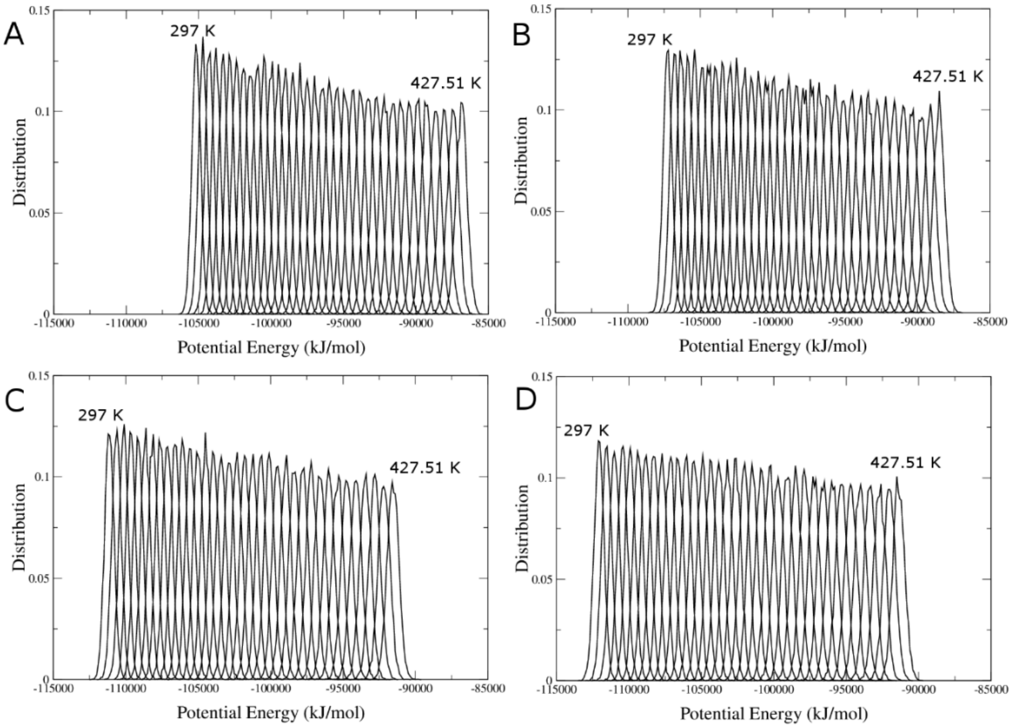

Supplementary Figure 26. Potential energy distributions of the replica exchange molecular dynamics (REMD) sampling for the DENV-2,4 fusion peptide (FP-1). Data is shown for: A) Amber99SB*-ILDN-Q, B) Charmm36, C) OPLSA-AA and D) Gromos54A7.

REFERENCES

1. Modis, Y., Ogata, S., Clements, D. & Harrison, S. C. A ligand-binding pocket in the dengue virus envelope glycoprotein. *Proc. Natl. Acad. Sci. U. S. A.* **100,** 6986–6991 (2003).

2. Modis, Y., Ogata, S., Clements, D. & Harrison, S. C. Structure of the dengue virus envelope protein after membrane fusion. *Nature* **427,** 313–319 (2004).

3. Zhang, Y. *et al.* Conformational changes of the flavivirus E glycoprotein. *Structure* **12,** 1607–1618 (2004).

4. Li, L. *et al.* The flavivirus precursor membrane-envelope protein complex: structure and maturation. *Science* **319,** 1830–1834 (2008).

5. Cockburn, J. J. *et al.* Structural insights into the neutralization mechanism of a higher primate antibody against dengue virus. *The EMBO Journal* **31,** 767–779 (2012).

6. Nayak, V. *et al.* Crystal structure of dengue virus type 1 envelope protein in the postfusion conformation and its implications for membrane fusion. *J. Virol.* **83,** 4338–4344 (2009).

7. Modis, Y., Ogata, S., Clements, D. & Harrison, S. C. Variable surface epitopes in the crystal structure of dengue virus type 3 envelope glycoprotein. *J. Virol.* **79,** 1223–1231 (2005).

8. Nybakken, G. E., Nelson, C. A., Chen, B. R., Diamond, M. S. & Fremont, D. H. Crystal structure of the West Nile virus envelope glycoprotein. *J. Virol.* **80,** 11467–11474 (2006).

9. Kanai, R. *et al.* Crystal structure of west nile virus envelope glycoprotein reveals viral surface epitopes. *J. Virol.* **80,** 11000–11008 (2006).

10. Cherrier, M. V *et al.* Structural basis for the preferential recognition of immature flaviviruses by a fusion-loop antibody. *EMBO J.* **28,** 3269–3276 (2009).

11. Luca, V. C., Nelson, C. A. & Fremont, D. H. Structure of the St. Louis encephalitis virus postfusion envelope trimer. *Journal of Virology* (2012). doi:10.1128/JVI.01950-12

12. Rey, F. A., Heinz, F. X., Mandl, C., Kunz, C. & Harrison, S. C. The envelope glycoprotein from tick-borne encephalitis virus at 2 A resolution. *Nature* **375,** 291–298 (1995).

13. Bressanelli, S. *et al.* Structure of a flavivirus envelope glycoprotein in its low-pH-induced membrane fusion conformation. *EMBO J.* **23,** 728–738 (2004).
